# Radical Decarboxylative Carbon–Nitrogen Bond Formation

**DOI:** 10.3390/molecules28104249

**Published:** 2023-05-22

**Authors:** Xiangting Li, Xiaobin Yuan, Jiahao Hu, Yajun Li, Hongli Bao

**Affiliations:** 1 College of Chemistry and Materials Science, Fujian Normal University, Fuzhou 350117, China; 2 Key Laboratory of Coal to Ethylene Glycol and Its Related Technology, State Key Laboratory of Structural Chemistry, Center for Excellence in Molecular Synthesis, Chinese Academy of Sciences, 155 Yangqiao Road West, Fuzhou 350002, China; 3 College of Chemistry, Fuzhou University, 2 Xueyuan Road, Fuzhou 350108, China

**Keywords:** C–N bond, carboxylic acids, decarboxylation, radical, redox–active, photocatalysis, metal catalysis

## Abstract

The carbon–nitrogen bond is one of the most prevalent chemical bonds in natural and artificial molecules, as many naturally existing organic molecules, pharmaceuticals, agrochemicals, and functional materials contain at least one nitrogen atom. Radical decarboxylative carbon–nitrogen bond formation from readily available carboxylic acids and their derivatives has emerged as an attractive and valuable tool in modern synthetic chemistry. The promising achievements in this research topic have been demonstrated via utilizing this strategy in the synthesis of complex natural products. In this review, we will cover carbon–nitrogen bond formation via radical decarboxylation of carboxylic acids, Barton esters, MPDOC esters, *N*–hydroxyphthalimide esters (NHP esters), oxime esters, aryliodine(III) dicarboxylates, and others, respectively. This review aims to bring readers a comprehensive survey of the development in this rapidly expanding field. We hope that this review will emphasize the knowledge, highlight the proposed mechanisms, and further disclose the fascinating features in modern synthetic applications.

## 1. Introduction

The main driving force for the tremendous developments in the area of C–N bond formation is that it allows the construction of diverse and ubiquitous nitrogen–containing compounds that are of significant importance in both small organic amines and complex azaheterocyclic systems. This is because many naturally existing organic molecules contain at least one nitrogen atom, such as amino acids, nucleic acids, and alkaloids. Moreover, nitrogen–containing compounds have also been universally applied in the synthesis of pharmaceuticals, agrochemicals, and functional materials. Consequently, the establishment of a powerful synthetic platform toward C–N bond formation will always be one of the major research areas in academia as well as in industry. Currently, chemists have the opportunity to use the well–developed toolbox for the construction of C(sp^2^)–N bonds, including Ullmann aminations [1,2], Chan–Lam aminations [3,4], and Buchwald–Hartwig reactions [5,6]. However, due to the feasible β–hydrogen elimination from the alkyl–metal intermediate and other deficiencies, developments on the construction of more challenging C(sp^3^)–N bonds by classical transition–metal–catalyzed reactions meet their limitation.

On the other hand, carboxylic acids are pervasive in nature. Owing to their stability, non–toxicity, and inexpensiveness, carboxylic acids can be regarded as ideal synthetic precursors and have actually been widely used in many named reactions, such as Kolbe electrolysis [7,8], the Ugi reaction [9], Curtius rearrangement [10,11], the Minisci reaction [12], the Corey–Nicolaou reaction [13,14], etc. In recent decades, radical decarboxylation of carboxylic acids and their derivatives has emerged as a powerful method for coupling reactions, largely due to the structural diversity of carboxylic acids and their capability to generate the C(sp^3^) radical site–specifically [15,16,17,18,19].

Pioneered by Barton in the 1980s [20,21,22], radical decarboxylative C–N bond formation was successfully realized using a series of *N*–hydroxy–2–thiopyridone esters, called Barton esters. Under light irradiation, the alkyl radical generated from Barton ester via decomposition would attack the N–N double bond of a diazirine to realize the formation of the C(sp^3^)–N bond. Two decades later, Renaud [23] developed another kind of alkylating reagent, the thiohydroxamate ester (MPDOC ester), for the light–driven C(sp^3^)–N_3_ bond formation. Notably, preactivation of carboxylic acids is inevitable for feasible transformations, and methodologies for C–N bond formation directly with carboxylic acids are eager to be developed. Through taking advantages of transition–metal catalysis, photocatalysis, electrocatalysis, and the emerged catalytic systems, many nitrogen–centered nucleophiles, including azides, amines, sulfonamides, carbamates, amides, nitriles, and so on, can participate the C–N bond formation reaction directly with carboxylic acids to afford valuable functionalized nitrogen–containing compounds. Later on, together with the great success with bench–stable *N*–hydroxyphthalamide esters (NHP esters), radical decarboxylative carbon–nitrogen bond formation has become a new avenue for C(sp^3^)–N bond formation, which is a great complement for conversional nucleophilic substitution (S_N_2) reactions, reduction of carbon–nitrogen double bonds, the Mitsunobu reaction [24], and others.

So far, reviews on C–N bond formation [25,26,27,28,29] and metal–catalytic [30,31,32,33,34,35,36,37,38] or photocatalytic [39,40,41,42,43,44] decarboxylation have been well reported. However, a comprehensive review about radical decarboxylation of carboxylic acids and their derivatives for the construction of carbon–nitrogen bonds is elusive. This review will focus on progress in radical decarboxylative C–N bond formation, discuss the mechanistic insights of these reactions, and emphasize synthetic applications. We hope that the important historical and recent developments discussed in this review will attract the attention of researchers both in organic synthesis and medicinal chemistry.

## 2. Radical Decarboxylative Coupling with Barton Esters

**Scheme 1:**
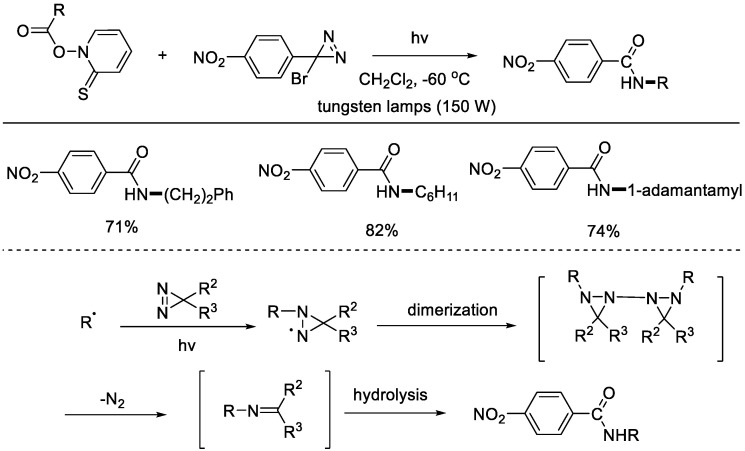
Light–driven radical decarboxylative C–N bond formation with Barton esters (Barton et al., 1993 [21]).

In 1993, the Barton group reported a light–initiated radical decarboxylative C–N bond formation with Barton esters (Scheme 1) [21]. The carbon source originated from the Barton esters, while 3–bromo–3–aryldiazirines were chosen as the carbon–radical captures. It was proposed that a dimer of the *N*–centered radical might be the intermediate, which then lost a molecule of nitrogen to form the imine intermediate. Further hydrolysis would afford the C–N coupling product.

**Scheme 2:**
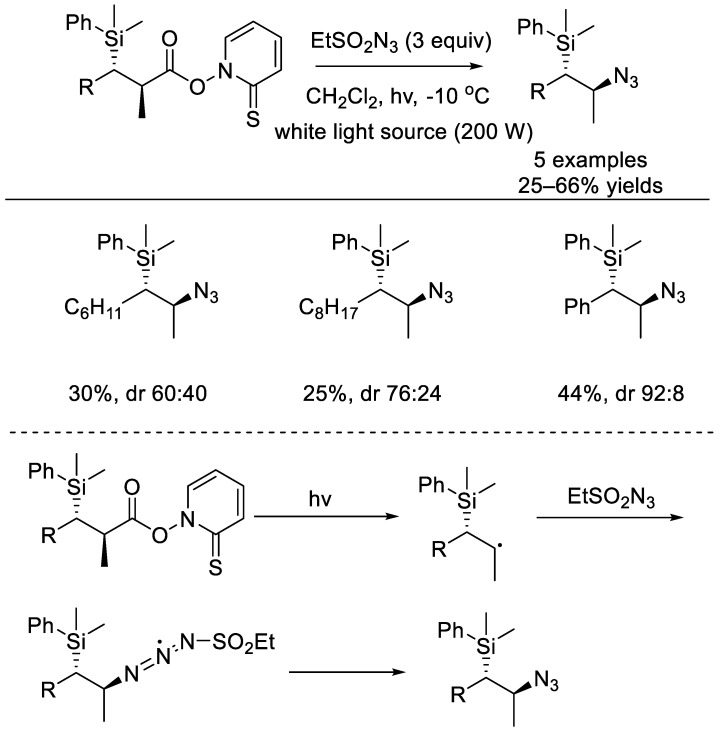
Light–driven radical decarboxylative C–N bond formation with Barton esters of β–silylcarboxylic acids (Porter et al., 2002 [22]).

In 2002, the Porter group reported that the Barton esters of β–silylcarboxylic acids underwent light–driven decomposition for the formation of C–N bond (Scheme 2) [22]. The β–silyl carbon radical generated would stereoselectively abstract the azido group from ethanesulfonyl azide.

## 3. Radical Decarboxylative Coupling with MPDOC Esters

**Scheme 3:**
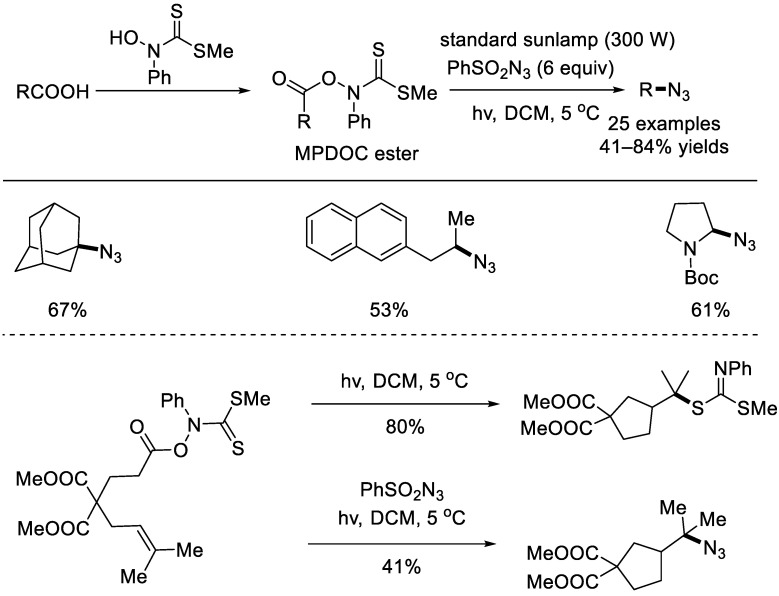
Radical decarboxylative azidation using MPDOC esters (Renaud et al., 2008 [23]).

In 2008, the Renaud group realized an efficient radical–mediated decarboxylative azidation of aliphatic carboxylic acids (Scheme 3) [23]. The success of this method lied in the use of thiohydroxamate esters (MPDOC esters). These MPDOC esters are more stable than the classical Barton esters. Initiated via light irradiation, the MPDOC ester would generate an alkyl radical, which abstracted the azido group from benzenesulfonyl azide to form the final alkyl azide. As shown below, in the absence of benzenesulfonyl azide, the reaction would also happen to afford the rearranged carbonimidodithioate in 80% yield.

## 4. Radical Decarboxylative Coupling with Carboxylic Acids

### 4.1. Transition–Metal–Catalyzed or Mediated Fashion

**Scheme 4:**
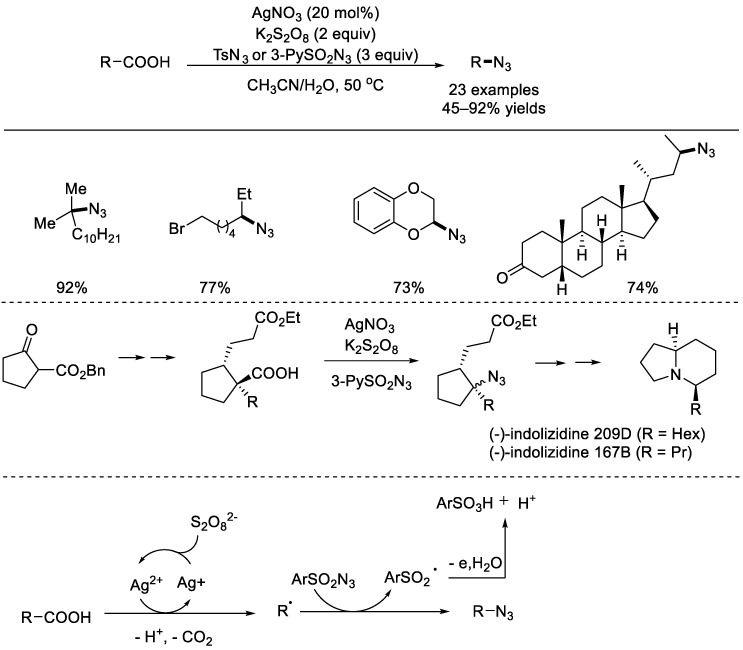
AgNO_3_–catalyzed decarboxylative azidations with aryl sulfonyl azides (Li et al., 2015 [45]).

In 2015, the Li group elegantly reported the first silver–catalyzed decarboxylative azidation of aliphatic carboxylic acids (Scheme 4) [45]. The reaction was performed in an aqueous solution and AgNO_3_ was used as the catalyst while K_2_S_2_O_8_ was used as the oxidant. A wide range of aliphatic carboxylic acids can react with toluenesulfonyl azide or pyridine–3–sulfonyl azide to afford the corresponding products. The successful synthesis of natural molecules (−)–indolizidine 209D and (−)–indolizidine 167B strongly emphasized the synthetic value of this method. A possible reaction mechanism was depicted. First, the Ag(I) species was oxidized with persulfate to produce Ag(II), an intermediate that grabbed an electron from the carboxylate to form the acyloxyl radical. The corresponding alkyl radical was generated via rapid decarboxylation. The alkyl azide will be available through azido group abstraction from the aryl sulfonyl azide.

**Scheme 5:**
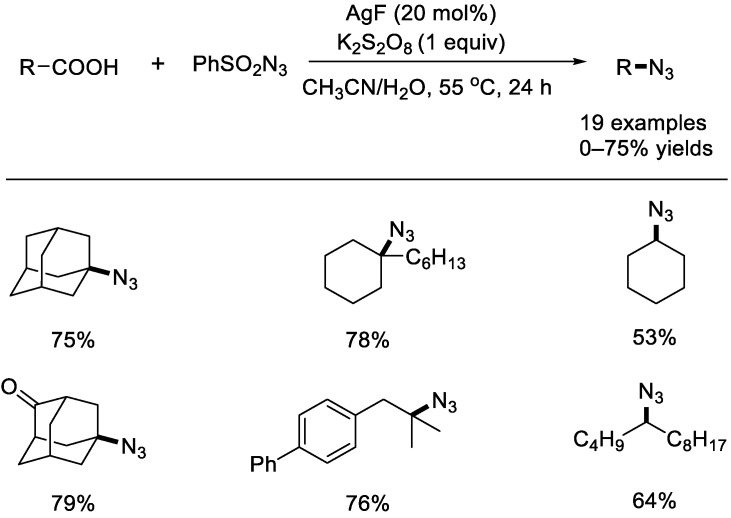
AgF–catalyzed decarboxylative azidations with aryl sulfonyl azides (Jiao et al., 2015 [46]).

Shortly after, the Jiao group found a similar silver–catalyzed decarboxylative azidation with aliphatic carboxylic acids (Scheme 5) [46]. A variety of tertiary, secondary, and primary organoazides can be produced from easily available aliphatic carboxylic acids. The EPR (electron paramagnetic resonance) experiments and DFT (density functional theory) calculations supported a radical mechanism.

**Scheme 6:**
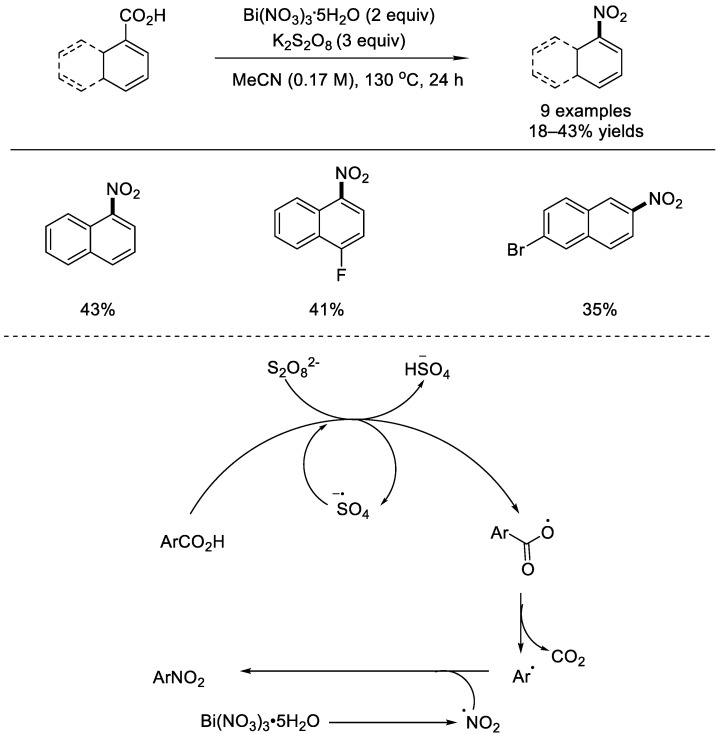
Bi(NO_3_)/K_2_S_2_O_8_ mediated transformation of aromatic carboxylic acids into nitroarenes (Maiti et al., 2019 [47]).

In 2019, the Maiti group described a novel strategy for selective access to nitroaromatics from carboxylic acids in an acid–free reaction conditions (Scheme 6) [47]. Bismuth nitrate was chosen as the source of the nitro source. Both aromatic and hetero–aromatic carboxylic acids were converted to nitroarenes. The authors outlined a possible radical mechanism. In the presence of S_2_O_8_^2–^, an aryl radical species would form in situ after H–atom abstraction and decarboxylation. Meanwhile, decomposition of the bismuth nitrate produced the nitro radical at an elevated temperature. The radical–radical cross–coupling afforded the corresponding nitro aromatics.

**Scheme 7:**
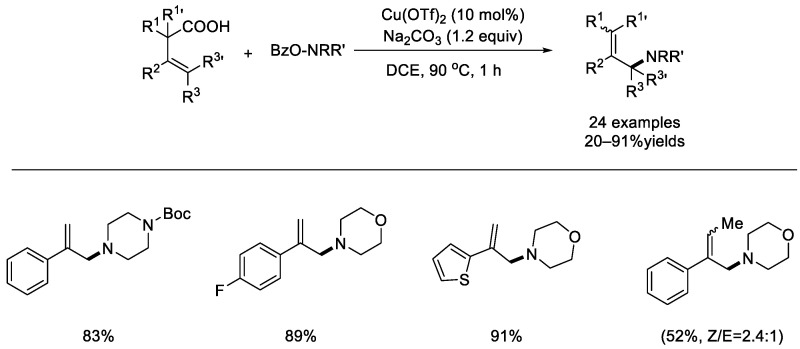
Copper–catalyzed decarboxylative functionalization of conjugated β,γ–unsaturated carboxylic acids (Wang et al., 2020 [48]).

In 2020, The Wang group reported a copper–catalyzed decarboxylative functionalization of conjugated β,γ–unsaturated carboxylic acids (Scheme 7) [48]. With Cu(OTf)_2_ as the catalyst, the reactions with *O*–acylzoyl–*N*–hydroxylamines afforded the corresponding allylic amination products in high yields. This method exhibited good functional group tolerance, straightforward operating process, and nice regioselectivity. The mechanism of this reaction may involve SET between the copper species and an *O*–acylzoyl–*N*–hydroxylamine, *N*–centered radical addition to conjugated β,γ–unsaturated carboxylic acids, and then decarboxylation via either a one–electron or two–electron pathway.

A similar work about copper–catalyzed decarboxylative functionalization of conjugated β,γ–unsaturated carboxylic acids via *N*–fluorobenzenesulphonimide (NFSI) was reported by the Fang group [49].

**Scheme 8:**
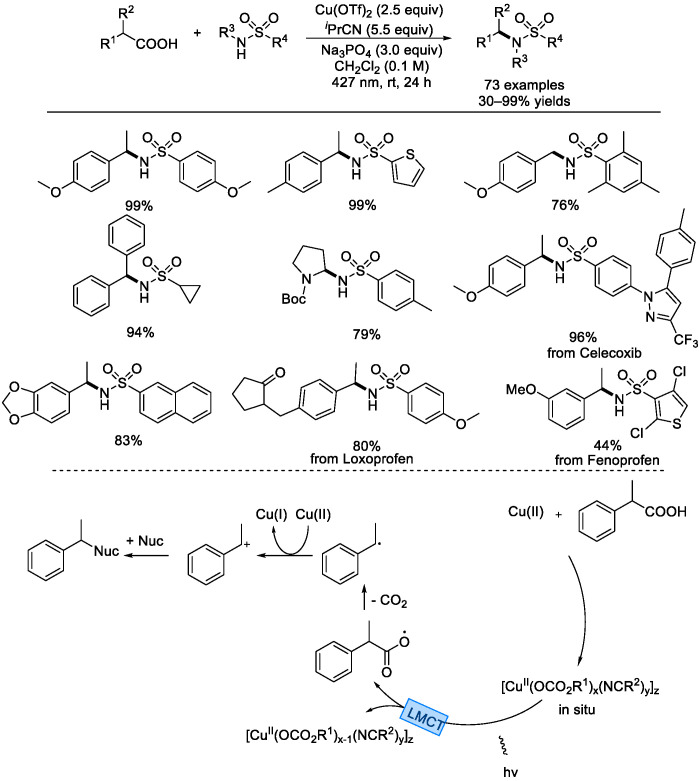
Radical decarboxylative cross–nucleophile coupling via ligand–to–metal charge transfer photoexcitation of Cu(II) carboxylates (Yoon et al., 2022 [50]).

In 2022, the Yoon group elegantly reported a decarboxylative cross–nucleophile coupling via ligand–to–metal charge transfer (LMCT) photoexcitation of Cu(II) carboxylates (Scheme 8) [50]. This net oxidative decarboxylation reaction worked well with a variety of substrates, including complicated medicinal compounds. Many carboxylic acids with diverse aryl functionalities can afford benzylic carbon radicals after radical decarboxylation. Various sulfonamide nucleophiles, carbamates, amides, and cyanides were good cross–nucleophiles for this C–N bond formation reaction. Based on mechanistic studies, this reaction might involve LMCT photoexcitation of a Cu(II) carboxylate complex assembled in situ, which homolytically decomposed to a carboxyl radical and the assembled Cu(I) carboxylate complex. Upon releasing the CO_2_ molecule, a benzylic carbon radical formed, which then was oxidized with the Cu(II) species to afford a benzylic carbocation. The final cross–coupling product would be generated via nucleophilic attack.

**Scheme 9:**
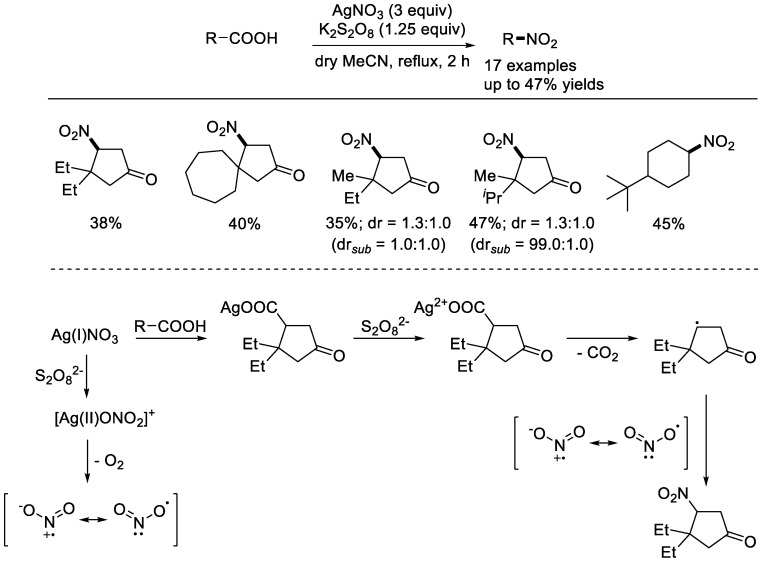
AgNO_3_/K_2_S_2_O_8_–mediated radical decarboxylative transformations of paraconic acids (Soorukram et al., 2022 [51]).

In 2022, the Soorukram group reported that the combined AgNO_3_/K_2_S_2_O_8_ system can be used for radical decarboxylative transformations of paraconic acids (Scheme 9) [51]. Under oxidative conditions, a range of paraconic acids can be used as the substrates, and a mixture of β–nitro and β–hydroxy γ–butyrolactones was produced. It is needed to mention that silver salt played a dual role in the reaction, both as the catalyst to activate the acids and as a source of the nitro group. Generally, the oxidation of carboxylic sliver using persulfate would lead to the formation of the alkyl radical. Meanwhile, the nitro radical was generated from the homolytic decomposition of the Ag(II)ONO_2_^+^ species. It was supposed that the final product was generated from the radical–radical coupling.

**Scheme 10:**
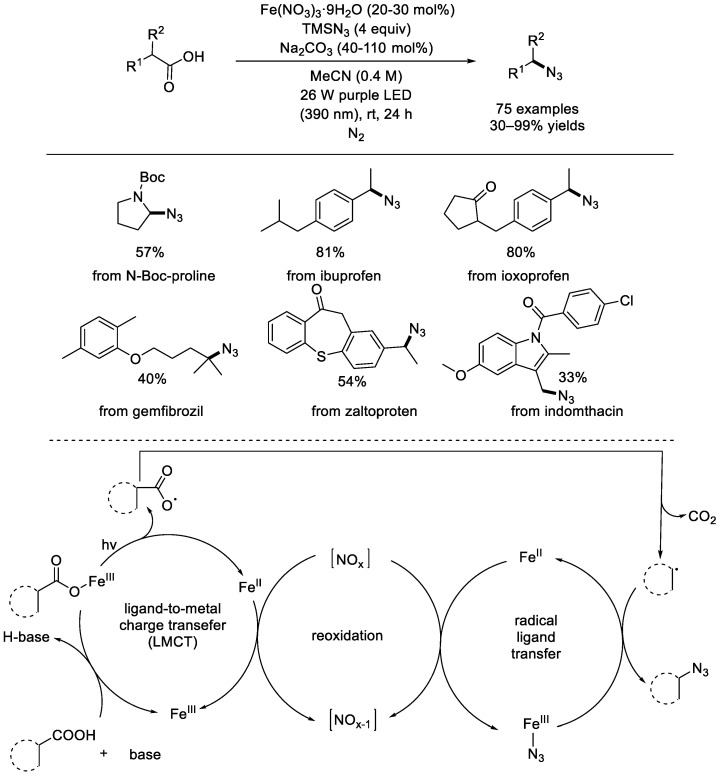
Photochemically iron–catalyzed decarboxylative azidation of carboxylic acids (West et al., 2023 [52]).

In 2023, the West group developed the first photochemically iron–catalyzed decarboxylative azidation reaction via the merged ligand–to–metal charge transfer and radical ligand transfer catalysis (Scheme 10) [52]. The reaction showed good tolerance for both electron–donating and electron–withdrawing functional groups, such as hydroxyl, hydroxymethyl, amino, phenylamino, difluoro, dichloro, bromine, trifluoromethyl, and methanesulfonyl groups. Based on mechanistic experiments, a possible mechanism of the iron–mediated photochemical, nucleophilic decarboxylative azidation, was proposed [53]. Firstly, in the presence of a base, ligand exchange between the carboxylic acid and Fe(III) species afforded the RCOO–Fe(III) species, which can undergo homolytic cleavage to produce a carboxyl radical and release the reduced iron(II) species through a photoinduced ligand–to–metal charge transfer process. Releasing the CO_2_ molecule created a carbon–centered radical, which underwent a radical ligand transfer (RLT) process mediated with the Fe–azide species to produce the azide product and the reduced iron(II) species. Nitrogen oxides played an important role in oxidation of iron(II) species into the trivalent iron species.

### 4.2. Photoredox or Dual Photoredox Transition–Metal–Catalyzed Fashion

**Scheme 11:**
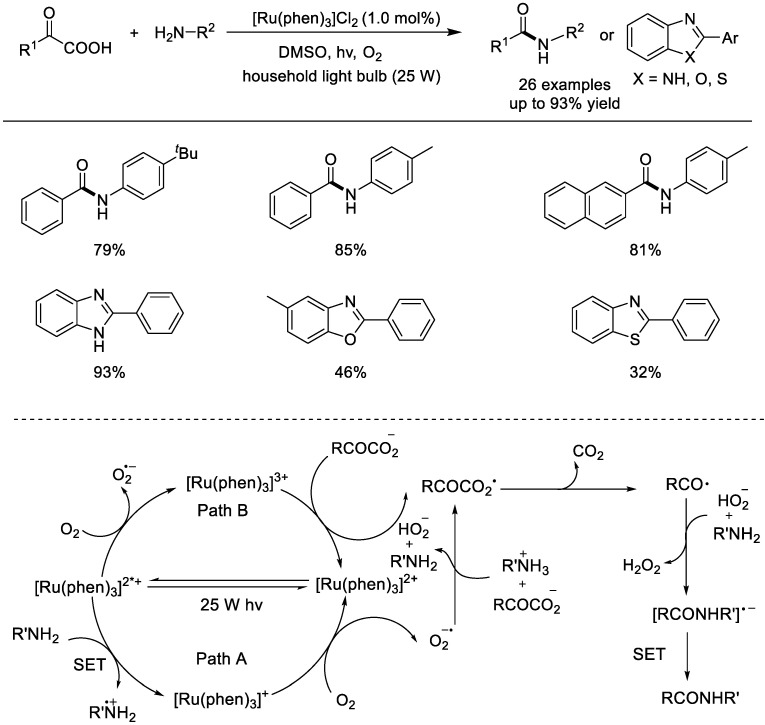
Photoredox Ru–catalyzed radical decarboxylation with α–keto acids. phen: 1,10–phenanthroline (Lei et al., 2014 [54]).

The decarboxylation reaction of α–keto acids with primary amines was reported by the Lei group in 2014 (Scheme 11) [54]. This is the first example of visible–light–mediated photocatalytic oxidative decarboxylation of α–keto acids. The authors evaluated the reaction parameters using benzoylphoric acid and 4–methylaniline as the reaction substrates and found that the reaction can be performed with [Ru(phen)_3_]Cl_2_ as the photocatalyst, O_2_ (balloon) as the oxidant, and DMSO as the solvent under 25 W fluorescent light irradiation. Many substituted α–keto acids can react with amines with electron–deficient or electron–rich substitutions and afford the corresponding products in high yields. Mechanistic studies, including electron paramagnetic resonance analysis, cyclic voltammetry (CV) experiments, a radical–trapping experiment, and density functional theory calculations, were performed. These mechanism studies revealed that (1) a SET process between the excited [Ru(phen)_3_]^2+^* and aniline was crucial, (2) the step of O_2_ activation might be the rate determining step, and (3) decarboxylation to form the aroyl radical was a fast and irreversible process.

A palladium–catalyzed decarboxylative amidation of α–keto acids with secondary amides [55] and a copper–catalyzed oxidative decarboxylative coupling of α–keto acids and sulfoximines [56] were also supposed to involve the aroyl radical intermediates.

**Scheme 12:**
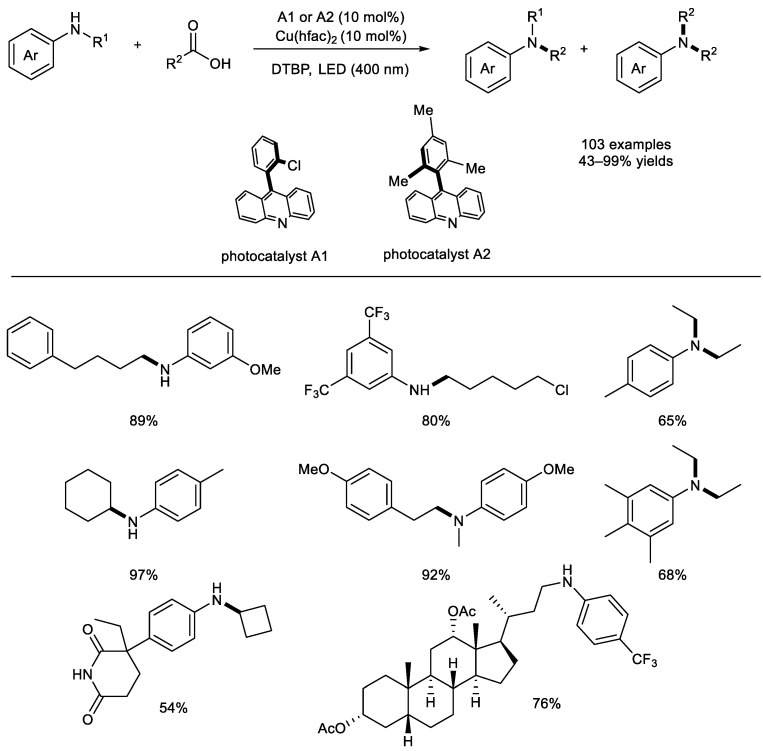
Visible–light–driven, acridine and copper co–catalyzed decarboxylation. hfac: hexafluoroacetylacetone; DTBP: di–tert–butyl peroxide (Larionov et al., 2020 [57]).

In 2020, the Larionov group reported a dual–catalytic method for the direct decarboxylative alkylation (DDA) of various aromatic carbocyclic and heterocyclic amines with carboxylic acids (Scheme 12) [57]. This visible–light–driven, acridine and copper co–catalyzed decarboxylation allowed the synthesis of *N*–alkylated secondary and tertiary anilines and *N*–heterocycles [58]. In addition, natural products and drugs underwent this reaction smoothly, and *N*–trideuteromethylation with AcOH–*d*_4_ could also work well. A range of carboxylic acids was also investigated; both primary carboxylic acids and the secondary alkyl carboxylic acids showed excellent reactivity. The EPR analysis, the Job plot, the Hammett plot, and the DFT computational study revealed that an acridine–catalyzed photoredox radical decarboxylation was involved in the reaction mechanism.

**Scheme 13:**
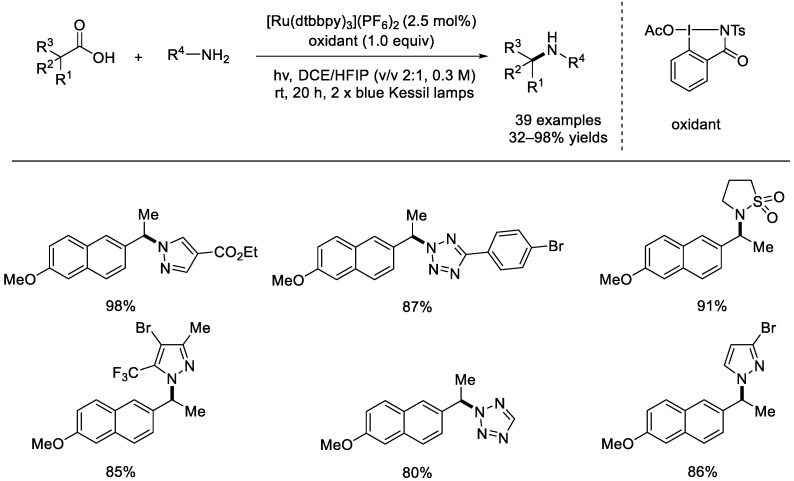
Photoredox Ru–catalyzed radical decarboxylative alkylation with benzylic carboxylic acids. DCE: 1,2–dichloroethane; HFIP: hexafluoroisopropanol (Terrett et al., 2021 [59]).

In 2021, the Terrett group reported a photoredox catalysis for decarboxylative alkylation of a range of nitrogen nucleophiles, including azoles, sulfonamides, ureas, and carbamates (Scheme 13) [59]. The optimal reaction conditions involved [Ru(dtbbpy)_3_](PF_6_)_2_ (dtbbpy = 4,4′–di–*tert*–butyl–2,2′–bipyridine) as the photocatalyst, an iodo(III) species as the oxidant, and DCE/HFIP as the mixed solvent. The transformation was also successful with a variety of benzylic carboxylic acids. A radical–polar crossover coupling was supposed to be promoted via the visible–light–mediated photo–redox process.

**Scheme 14:**
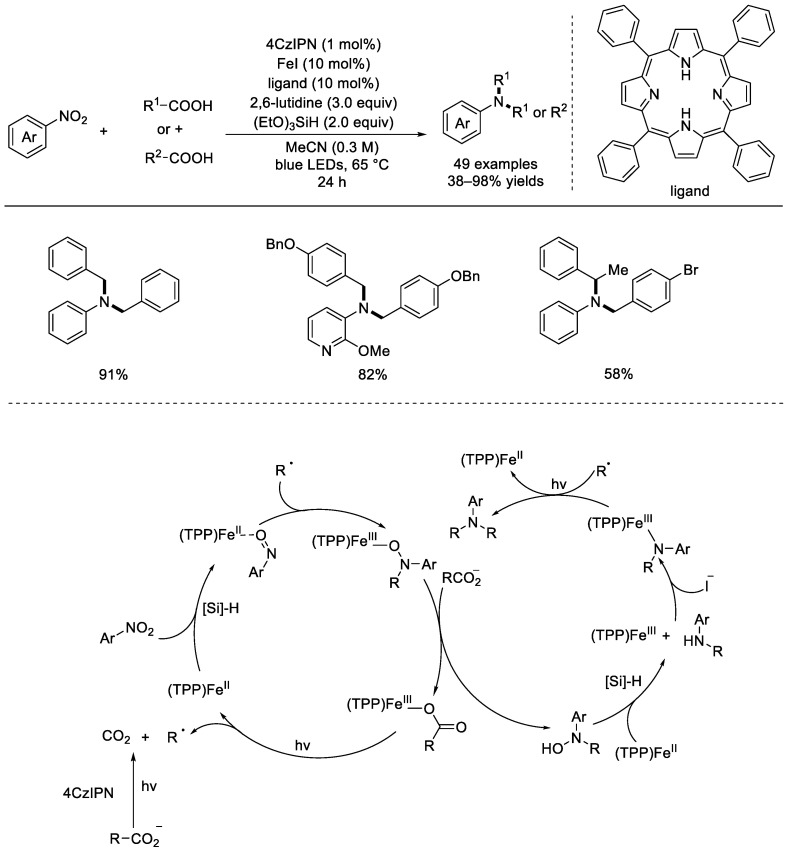
4CzIPN photocatalyzed radical tandem C–N coupling of nitroaromatics and carboxylic acids. 4CzIPN: 2,4,5,6–tetrakis(carbazol–9–yl)–1,3–dicyanobenze (Xie et al, 2022 [60]).

In 2022, the Xie group developed a radical tandem C–N coupling strategy for the efficient construction of aromatic tertiary amines from commercially available carboxylic acids and nitroarenes (Scheme 14) [60]. 4CzIPN was used as the photocatalyst, FeI_2_ was chosen as the metal catalyst, and (EtO)_3_SiH was found to be the best reductive agent. Under light irradiation, many affordable carboxylic acids and nitroaromatics can undergo effectively this decarboxylative C–N coupling. Notably, two different carboxylic acids would allow the concise synthesis of nonsymmetric aromatic tertiary amines in fair yields. An S_H_2 (bimolecular homolytic substitution) pathway might be critical in the C–N bond formation step.

**Scheme 15:**
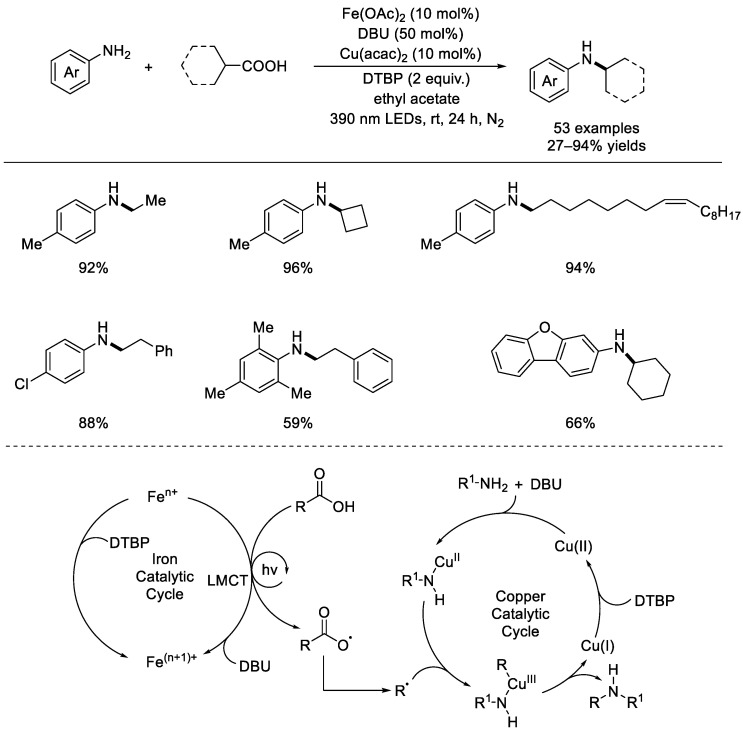
Dual photoinduced iron–catalyzed and copper–catalyzed decarboxylative amination. DBU: 1,8–diazabicyclo [5.4.0] undec–7–ene; acac: acetylacetone (Zeng et al., 2023 [61]).

In 2023, the Zeng group reported a dual photoinduced iron–catalyzed and copper–catalyzed decarboxylative amination (Scheme 15) [61]. Aliphatic carboxylic acids and arylamines were used as starting materials for the radical decarboxylative functionalization. A series of primary, secondary, and tertiary carboxylic acids was screened to allow the preparation of corresponding alkyl amines in great efficiencies. Significantly, in addition to the various functional groups, such as halogen, CF_3_, ester group, carbonyl group, and amide being tolerated, some natural molecules containing the carboxylic acid group can also obtain amination products in good yields. Besides, at the para–position of the aromatic amines, electron–donating groups (MeO, *^t^*Bu, PhO, and PhS) and electron–withdrawing groups (Cl and Br) can be presented, and the required products can be produced in moderate to excellent yields. Based on the results of the mechanistic experiments, a catalytic reaction sequence was proposed. Firstly, Fe(II) was oxidized to trivalent Fe(III) via DTBP. Then, the ligand–to–iron charge transfer occurred under an irradiation of light to generate RCOO·, and releasing the CO_2_ molecule subsequently obtained the alkyl radical R·. On the other hand, in the presence of DBU, ligand exchange with an aromatic amine formed an amino Cu(II) species, which then rapidly trapped the alkyl radical to afford an alkyl amino copper(III) species. Reductive elimination formed the C–N bond. DTBP was proven to be the proper oxidant for regeneration of the Cu(II) species as well as the Fe(II) species.

### 4.3. Electrochemical Fashion

**Scheme 16:**
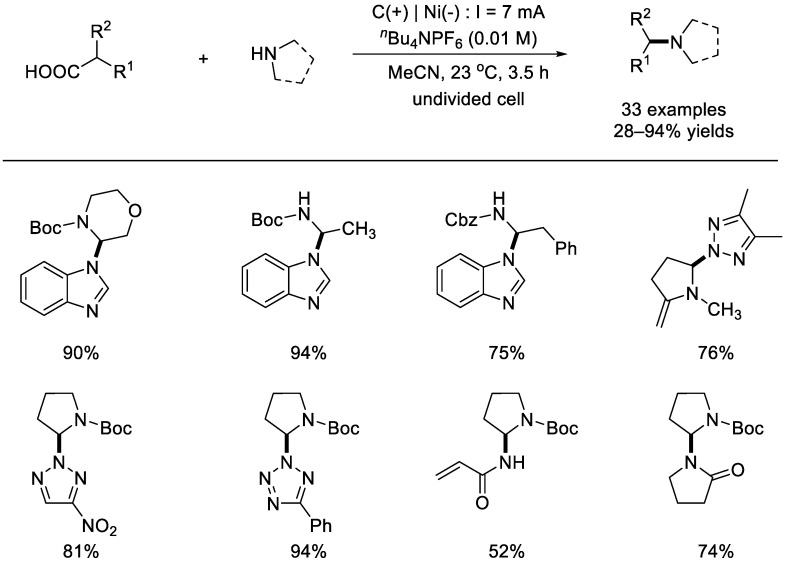
Electrochemical oxidation of amino acids for decarboxylative C(sp^3^)–N couplings (Wang et al., 2019 [62]).

In 2019, the Wang group reported an electrochemical oxidation of amino acids for the development of decarboxylated C(sp^3^)–N coupling reactions (Scheme 16) [62]. The reactions were performed in acetonitrile at room temperature through using a constant current of 7 mA in an undivided cell equipped with a graphite anode and a nickel cathode. A wide range of amino acids, including proline, acetidine carboxylic acids, hexameric α–amino acids, and cyclic *N*–protected–amino acids can undergo anodic oxidative decarboxylation. Nucleophilic reagents such as pyrazoles, 1,2,3– and 1,2,4–triazoles, benzotriazoles, and tetrazoles were found to be effective. These results implied that the C–N coupling process of decarboxylation via electrochemical oxidation was a useful complement to photo–redox catalysis. Mechanistically, the amino acid was supposed to be oxidized into a stabilized carbon radical, which was further oxidized to become the stabilized carbocation.

**Scheme 17:**
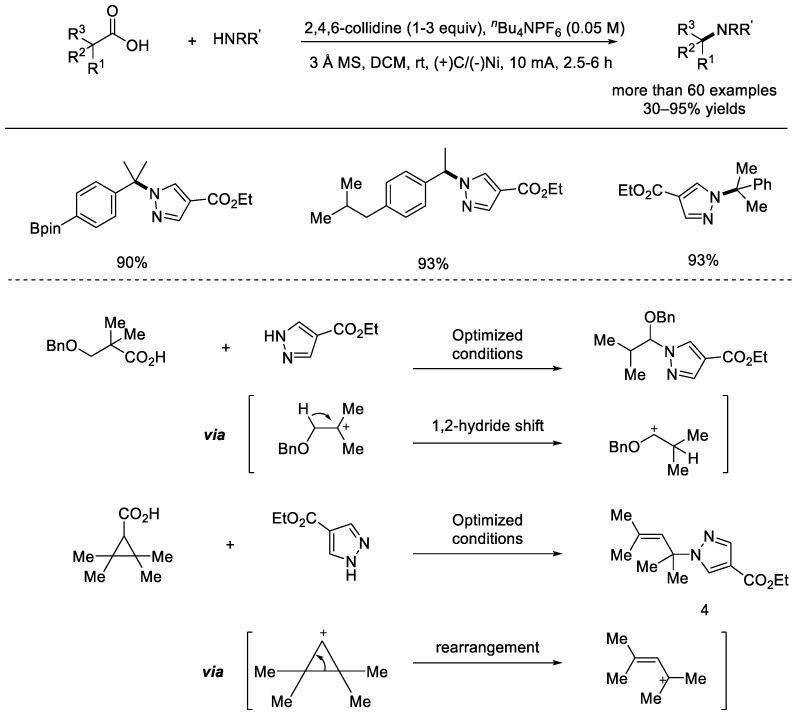
Electrochemical decarboxylation *N*–alkylation of heterocycles with carboxylic acids. MS: molecular sieve (Baran et al., 2020 [63]).

In 2020, the Baran group disclosed the electrochemical decarboxylation *N*–alkylation of heterocycles (Scheme 17) [63]. Many non–activated carboxylic acids were used as alkylating agents and heterocycles were applied as the nitrogen sources in the *N*–alkylation of heterocycles through utilizing an electrochemically powered anodic decarboxylation process. Various functional groups such as ester group, cyano group, BPin, nitro group, acetal, halide, and fluoroalkyl groups were tolerated. Therefore, practical synthesis of pharmaceuticals and agrochemicals with this method was predicted. Mechanistically, it was proposed that the decarboxylation would result in the formation of cationic intermediates, as evidenced by the formation of 1,2–hydride shift product and the product from cationic rearrangement.

**Scheme 18:**
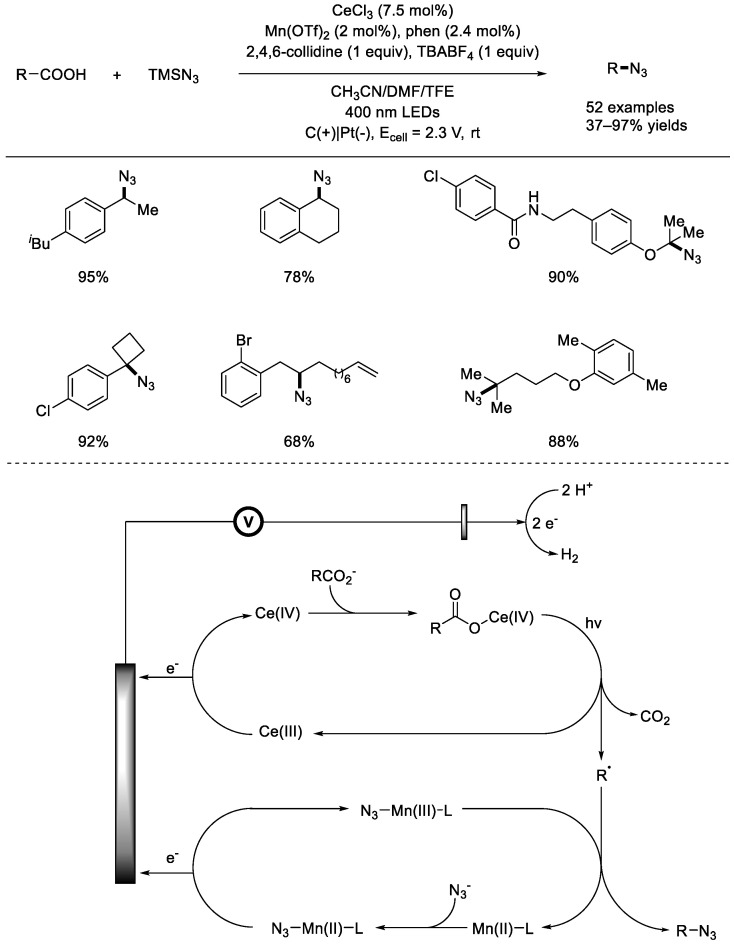
Electrophotochemical metal–catalyzed radical decarboxylative functionalization of aliphatic carboxylic acids. TBABF_4_: ammonium tetra–*n*–butyl tetrafluoroborate (Fu et al., 2022 [64]).

In 2022, the Fu group reported an electrophotochemical metal–catalyzed radical decarboxylative functionalization of aliphatic carboxylic acids under mild reaction conditions (Scheme 18) [64]. A diverse array of aliphatic carboxylic acids, including arylacetic acids; α–oxy and amino carboxylic acids; primary, secondary, and tertiary carboxylic acids; and natural carboxylic acids, can be applied for the generation of corresponding alkyl azides. Based on the radical probe experiment and cyclic voltammetry studies, a possible mechanism for this electrophotochemical metal–catalyzed C–N formation was proposed. While electrochemistry promoted the oxidation of Mn(II)–N_3_ into Mn(III)–N_3_ and Ce(III) into Ce(IV), photochemistry facilitated the LMCT of the Ce(IV)–OOCR to generate the alkyl radical.

### 4.4. Metal–Free Fashion

**Scheme 19:**
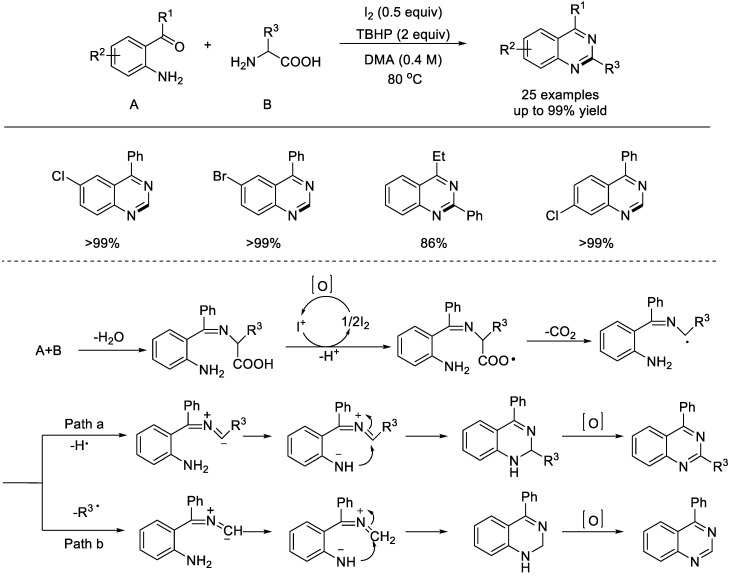
Metal–free decarboxylative coupling of 2–amino benzophenones and primary α–amino acids. DMA: dimethylacetamide (Wang et al., 2011 [65]).

In 2011, the Wang group reported an intramolecular oxidative decarboxylative amination of primary α–amino acids under metal–free conditions (Scheme 19) [65]. The reaction with 2–amino benzophenones and phenylalanines can be performed with molecular iodine as the catalyst and *tert*–butyl hydrogen peroxide (TBHP) as the oxidant. A variety of 2–amino benzophenones and primary α–amino acids underwent the oxidative decarboxylation couplings to afford the desired quinazolines. This decarboxylative C–N coupling showed many advantages, such as metal–free conditions, low toxicity, water and air tolerance, and environmental friendliness. The authors suggested that this oxidative decarboxylation coupling may involve a radical mechanism. The condensation of 2–amino benzophenone and a primary α–amino acid resulted in the initial formation of an imine, which upon single–electron transfer (SET) oxidation using an I^+^ species will produce an acyloxy radical. After releasing a mole of CO_2_, the alkyl radical was generated. There are two possible pathways: (a) hydrogen atom abstraction and (b) R^3^ radical removing. Through the same cascade process of 1,6–proton transfer, intramolecular cyclization, and oxidation, quinazoline product will be produced.

**Scheme 20:**
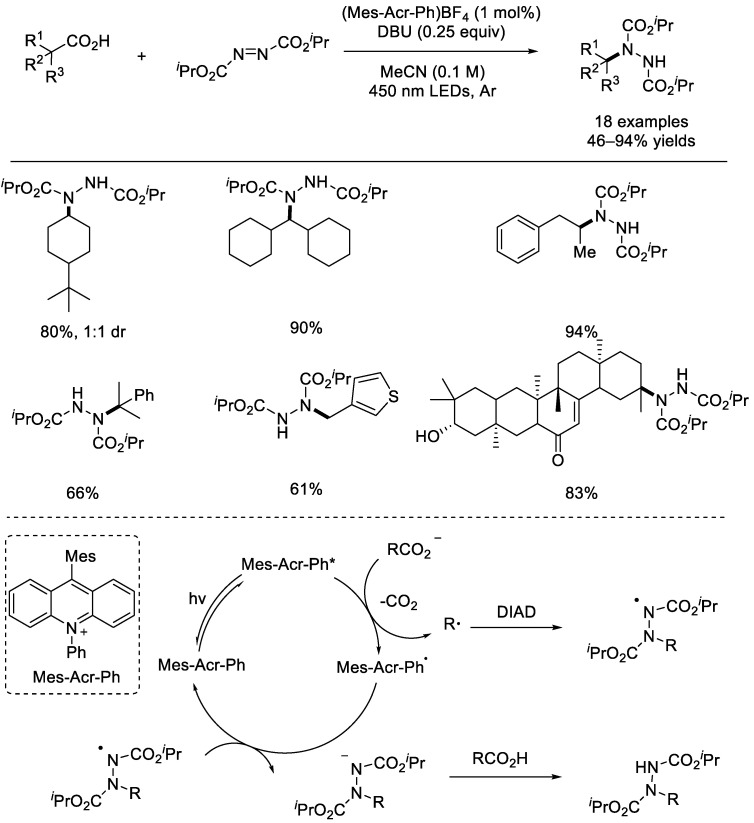
Mes–Acr–Ph–catalyzed reaction of DIAD with saturated aliphatic acids (Tunge et al., 2016 [66]).

In 2016, the Tunge group developed an aminodecarboxylation of unactivated alkyl carboxylic acids utilizing an organic photocatalyst. Diisopropyl azo–dicarboxylate (DIAD) was chosen to forge the desired C–N bond, while Mes–Acr–Ph (9–mesityl–10–methylacridinium) BF_4_ was selected as the photocatalyst (Scheme 20) [66]. A wide range of alkyl carboxylic acids can be used in this reaction for the site–selective formation of amination products. A possible mechanism was proposed based on the preliminary mechanistic studies. First, the photocatalyst Mes–Acr–Ph was subjected to light irradiation to generate the excited Mes–Acr–Ph*, which can oxidize the alkyl carboxylate to produce the alkyl radical upon releasing a molecule of CO_2_. Then, after trapping via DIAD, a *N*–center radical was formed, which was reduced using Mes–Acr–Ph^•^ to afford the corresponding amide anion. The ground state photocatalyst Mes–Acr–Ph would be regenerated from Mes–Acr–Ph^•^.

**Scheme 21:**
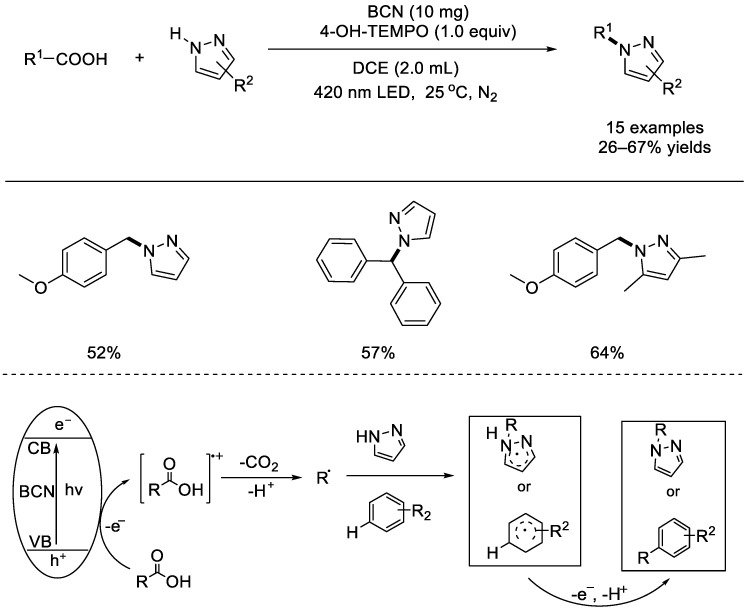
Boron carbon nitride complex–mediated radical decarboxylative C(sp^3^)–N bond (Wang et al., 2021 [67]).

In 2021, the Wang group reported a direct radical decarboxylative C(sp^3^)–N bond formation with carboxylic acids catalyzed by metal–free boron carbon nitride (BCN) complex (Scheme 21) [67]. In the absence of strong oxidants, bases, or transition metals, the ceramic BCN semiconductor demonstrated good photocatalytic performance for direct decarboxylation. Many arylacetic acids with electron–rich substituents, such as methoxy, methyl, and *tert*–butyl groups, were easily transformed into the desired products. The technology was appropriate for gram–scale and late functionalization of medicinal compounds. This heterogeneous catalysis can be recycled and reused without losing reactivity. Initially, the photo–generated hole at the VB (valence band) of BCN oxidized the aryl acetic acid to form a radical carbocation intermediate, which would afford the alkyl radical upon rapid decarboxylation. The carbon–centered radical was then intercepted by a pyrazole or a (hetero)arene, thus forming the corresponding product, finally.

## 5. Radical Decarboxylative Coupling with NHP Esters

*N*–hydroxyphthalamide esters (NHP esters) derived from carboxylic acids have received much attention as redox–active esters from organic chemists. In this section, we will discuss the radical decarboxylative C–N bond formation with NHP esters.

**Scheme 22:**
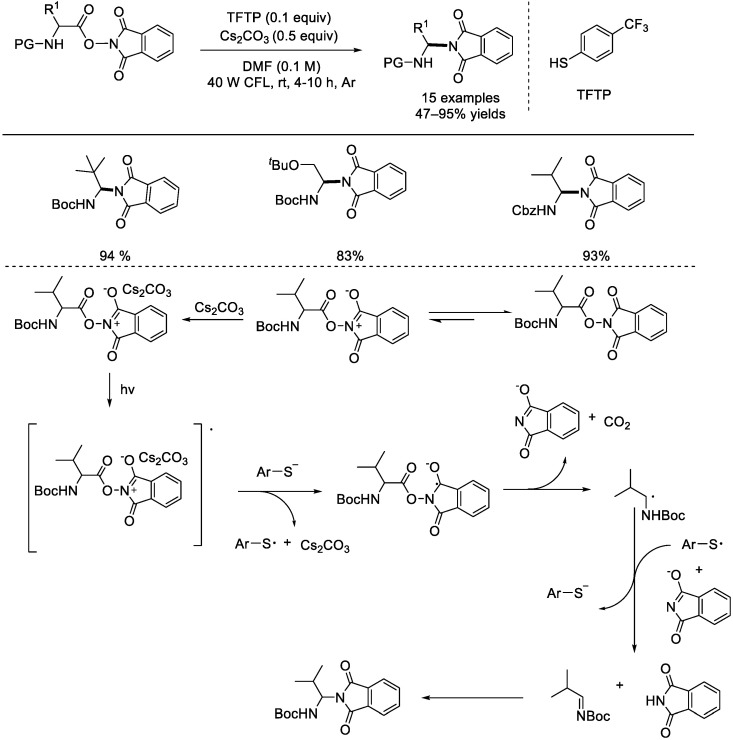
Photoredox decarboxylative couplings under thiophenol catalysis. CFL: compact fluorescent lamp (Fu et al., 2016 [68]).

In 2016, the Fu group reported a photoredox decarboxylative coupling under thiophenol catalysis (Scheme 22) [68]. It was found that decarboxylation of NHP esters can be done without the use of any photocatalyst, and 4–(trifluoromethyl)thiol showed the best catalytic activity. Under the optimal reaction conditions, many amino–acid–derived NHP esters reacted smoothly to provide the corresponding products in good yields. The complex of NHP ester and the base Cs_2_CO_3_ was initially activated via light irradiation; the activated species would undergo a SET process with an ArS^−^ anion to afford the ArS^•^ radical and the radical anion species that afforded the alkyl radical upon fragmentation. After oxidation via ArS^•^ radical, the alkyl radical was transformed into an imine. Ultimately, the target product was produced via nucleophilic addition.

**Scheme 23:**
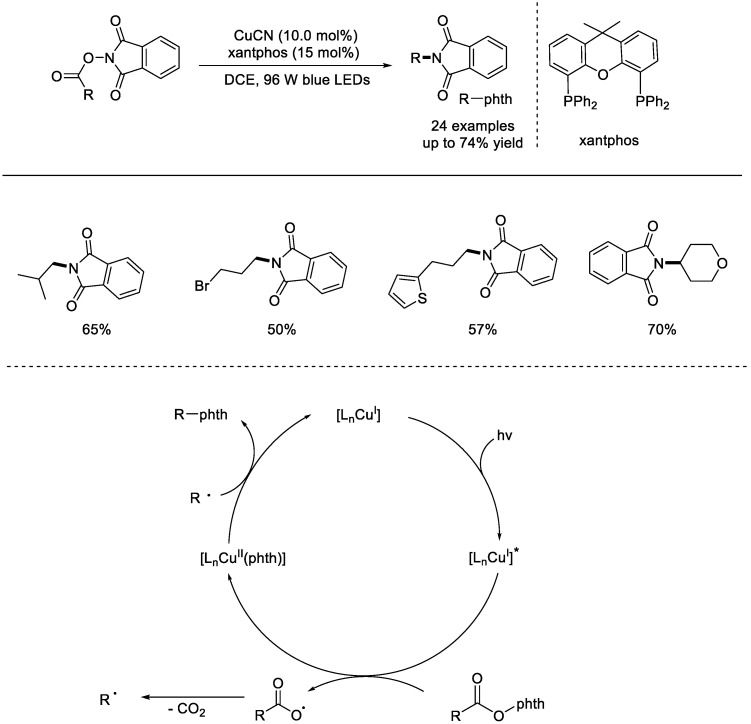
Light–driven, copper–catalyzed decarboxylation coupling of NHP esters (Fu et al., 2017 [69]).

In 2017, the Fu group reported decarboxylation of NHP esters using a copper catalyst in combination with blue LED (light emitting diode) irradiation (Scheme 23) [69]. This method was regarded as an excellent alternative to the classic and powerful Curtius rearrangement. The conditions of this reaction were mild, and a variety of alkyl carboxylic acids were well–compatible. Functional groups such as olefins, halogens, ethers, esters, and ketones were all tolerated. Based on the results of radical trapping and radical clock experiments, a possible reaction mechanism was proposed. Ligated copper was excited under the activation of light. The excited copper complex can reduce the NHP esters through a SET process and generate the copper(II) species and a radical anion intermediate. Upon fragmentation, an alkyl radical would be formed, which then reacted with the copper(II) species to generate the target compound.

**Scheme 24:**
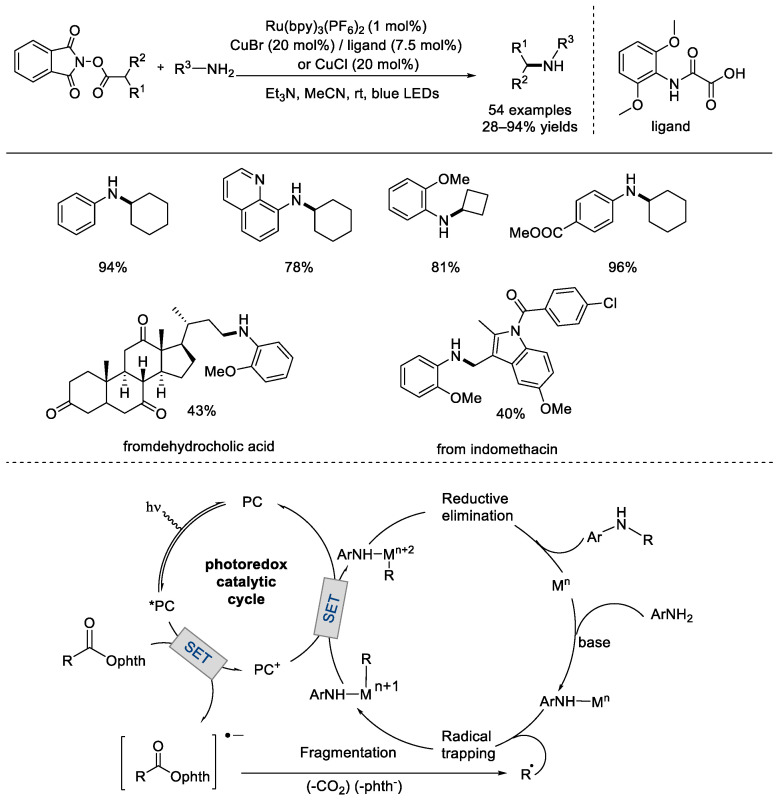
Ru–photocatalyst and copper catalyst co–realized decarboxylation coupling reaction with additional amines (Hu et al., 2018 [70]).

In 2018, the Hu group achieved the radical decarboxylative coupling of NHP esters with extra amine additives using a dual catalytic system of photocatalyst and copper catalyst (Scheme 24) [70]. The transformation can be used for the conversion of primary and secondary carboxylic acids and for the functionalization of amino acids, natural products, and pharmaceutical molecules. Various alkylated amines, including those in pharmaceutical and materials science, can be obtained from this C–N coupling reaction. Mechanistically, the photocatalyst was excited using visible light to produce an excited–state complex that reduced the redox–active ester via single–electron transfer. The reduced ester was then decarboxylated, thus yielding an alkyl radical and a molecule of CO_2_. In the other cycle, ligand exchange between the transition metal and an amine generated the low–valent metal amido complex, which trapped the alkyl radical to form an intermediate complex bonded with the amine and the alkyl. A single–electron transfer from the intermediate to the oxidized photocatalyst completed the photocatalytic cycle and yielded a high–valent metal alkyl amido complex as well. The desired amine product was then produced via C–N reductive elimination, which also regenerated a low–valent metal species.

**Scheme 25:**
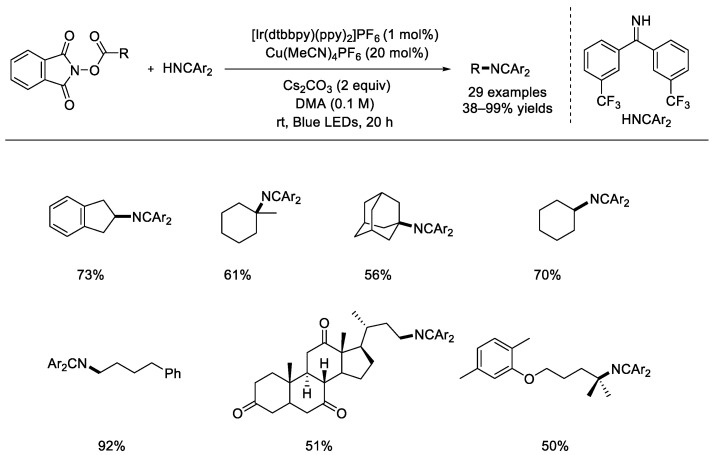
Ir–photocatalyst and copper catalyst co–realized decarboxylation coupling reaction with additional amines (Hu et al., 2018 [71]).

The Hu group published a similar radical decarboxylative C(sp^3^)–N formation with NHP esters (Scheme 25) [71]. Ir[(dtbbpy)(ppy)_2_]PF_6_ was used as the photocatalyst and Cu(MeCN)_4_PF_6_ was selected as the transition–metal catalyst. Through taking advantage of benzophenone imine as the nitrogen source, this method can be used for the synthesis of primary alkylamines.

**Scheme 26:**
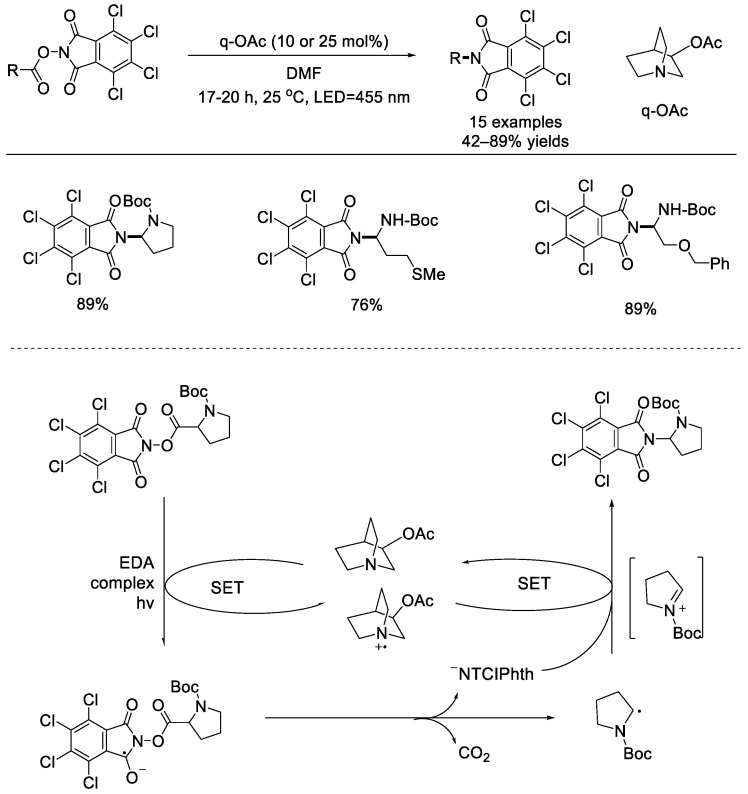
3–Acetoxyquinuclidine catalyzed radical decarboxylation for C–N bond formation (Bach et al., 2019 [72]).

In 2019, the Bach group reported a metal–free radical decarboxylation of C–N bond formation with 3–acetoxyquinuclidine (q–OAc) as the electron donor catalyst (Scheme 26) [72]. The electron donor–acceptor (EDA) complexes of redox–active ester and 3–acetoxyquinuclidine can be activated using visible light. Both α–amino acids and α–hydroxy acids derivatives can react smoothly to afford the corresponding C–N bond formation products in good to high yields. These results demonstrated that q–OAc can be used as a reversible SET catalyst. The reaction began with the light–initiated SET between the NHP ester and q–OAc. Followed by decomposition of the radical anion, an α–amino–stabilized carbon radical can be generated, which can then be oxidized using q–OAc^+•^ to afford the iminium ion. Nucleophilic attack from the previously liberated tetrachlorophthalimide anion would deliver the observed product.

**Scheme 27:**
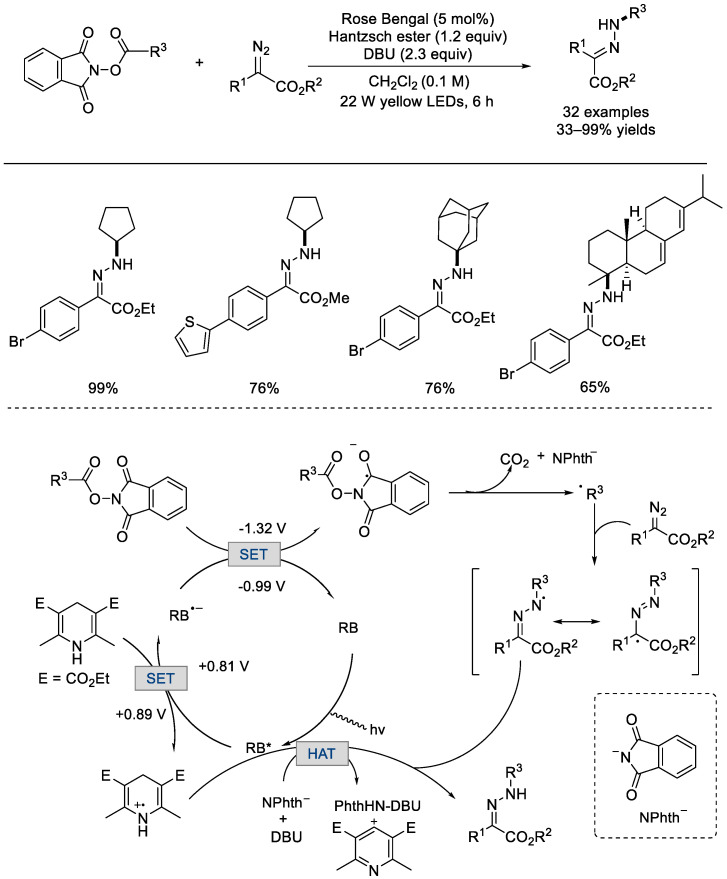
Rose Bengal photocatalyzed *N*–alkyl hydrazones formation from α–diazoacetates and NHP esters (Yu et al., 2019 [73]).

In 2019, the Yu group achieved the synthesis of structurally and functionally distinct *N*–alkyl hydrazones from α–diazoacetates and NHP esters via a metal–free photocatalytic coupling process (Scheme 27) [73]. More than 60 inaccessible C(sp^3^)–rich *N*–alkylhydrazones were produced utilizing Rose Bengal as the photocatalyst under yellow LED irradiation. The fluorescence quenching analysis and deuterium incorporation studies revealed that the Hantzsch ester played a significant role in the process. The reaction cycle began with the photoexcitation of Rose Bengal (RB). The RB* would undergo single–electron transfer with the Hantzsch ester to produce the radical anion (RB^•−^) [E(RB/RB^•−^) = 0.99 V versus SCE (saturated calomel electrode)], which could reduce the NHP ester to form RB and a radical anion species. Followed by C–C bond fragmentation, an alkyl radical was generated. The C(sp^3^)–N bond would then be formed as a result of coupling of the alkyl radical with the diazo compound. The hydrazone product would be afforded followed by hydrogen–atom abstraction from the cationic radical Hantzsch ester.

**Scheme 28:**
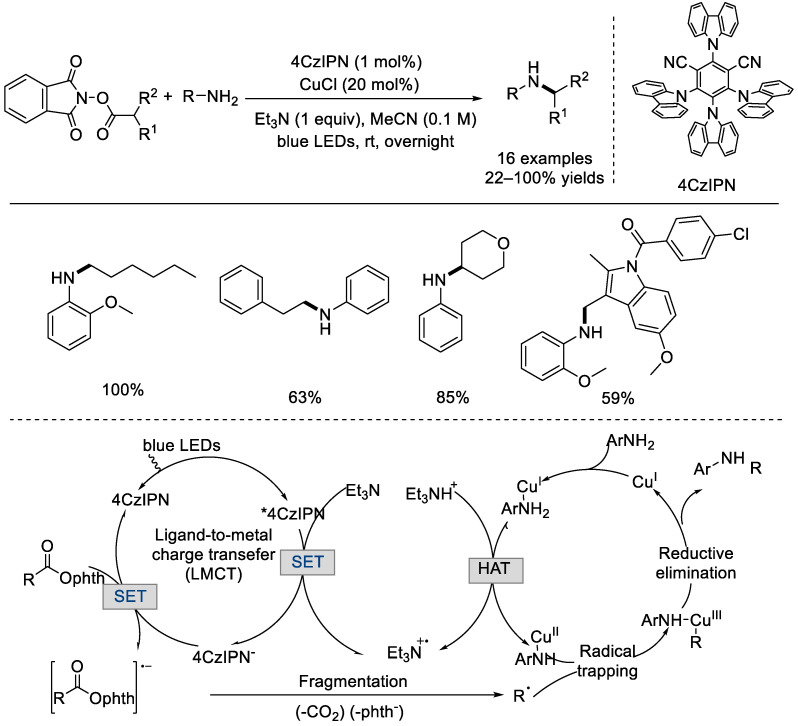
4CzIPN and CuCl co–catalyzed decarboxylative C(sp^3^)–N couplings (Hu et al., 2020 [74]).

In 2020, the Hu group developed an organic photocatalyst, 4CzIPN, and a copper catalyst, CuCl, which co–catalyzed decarboxylative C(sp^3^)–N couplings (Scheme 28) [74]. Under light irradiation, a variety of anilines and imines coupled with primary and secondary alkyl radicals to afford the desired amines in good to high yields. A reductive quenching process and tandem copper catalysis were proposed as the mechanism.

**Scheme 29:**
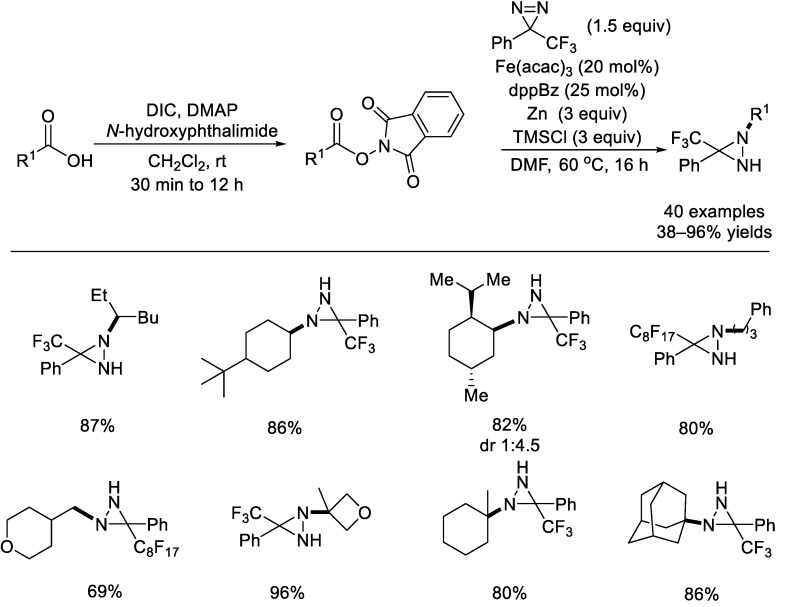
Iron–catalyzed radical decarboxylative C(sp^3^)–N formation with diazirines. DIC: *N*, *N*–diisopropylcarbodiimide; DMAP: 4–dimethylaminopyridine; dppBz: 1,2–bis(diphenylphosphanyl)benzene (Lopchuk et al., 2020 [75]).

In 2020, the Lopchuk group investigated an iron–catalyzed radical decarboxylative C(sp^3^)–N formation through the use of diazirines as practical nitrogen reagents and both simple and complex redox–active esters as the alkyl radical precursors (Scheme 29) [75]. A variety of primary, secondary, and tertiary alkyl carboxylic acids reacted well and the trifluoromethyl group, carbamates, alcohols, ketones, sulfones, ethers, esters, enones, olefins, and lactones were all tolerated. The corresponding diaziridine products can be selectively hydrolyzed, leaving either one or both nitrogen atoms on the substrates. Moreover, these diaziridines can be synthetically applied to the construction of pharmaceutically relevant building blocks. Fluorous phase synthesis can be used to produce perfluorinated diazines in both steps of the amination process, enabling the creation of high–throughput libraries of nitrogen–rich molecules.

**Scheme 30:**
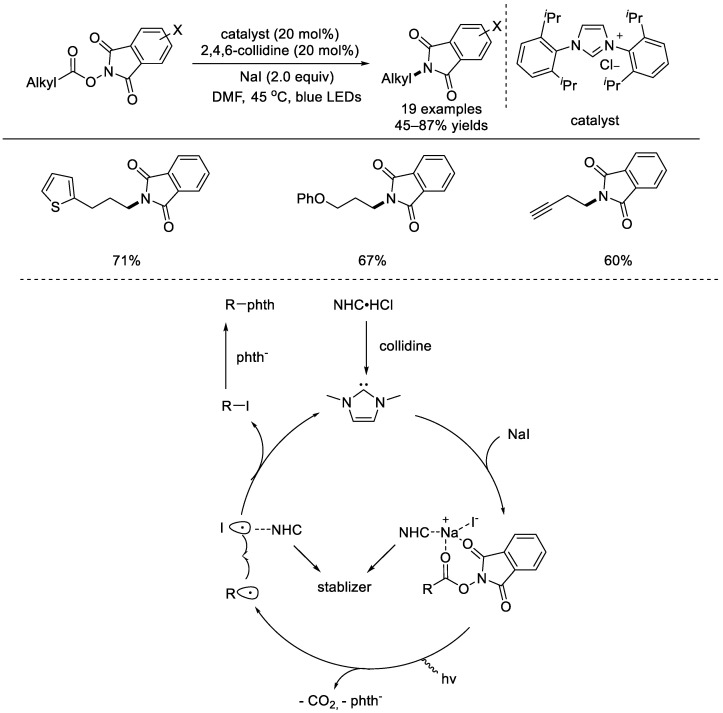
Light–driven, NHC–catalyzed radical decarboxylative C–N bond formation (Chen et al., 2020 [76]).

In 2020, the Chen group applied an electron donor–acceptor (EDA) strategy for radical decarboxylative C–N bond formation (Scheme 30) [76]. The NHP ester–NaI EDA complex can be activated upon light irradiation and coupling products, such as primary C(sp^3^)–(N)phth, secondary C(sp^3^)–I, and tertiary C(sp^3^)–(meta C)phth can be selectively afforded. The primary C(sp^3^)–(N)phth was supposed to obtained from S_N_2 substitution reactions between the resulting iodoalkanes and the in–situ–generated phthalimide anions. The substrate scope was broad and the functional group tolerance was fair. First, an EDA complex formed from the NHP ester and sodium iodide, which was stabilized with the NHC catalyst. The photoactive EDA complex conducted single–electron transfer to create the alkyl radical, phthalimide anion, and iodine atom stabilized with NHC. Radical coupling generated the alkyl iodide, which reacted with the phthalimide anion to form the C–N bond.

**Scheme 31:**
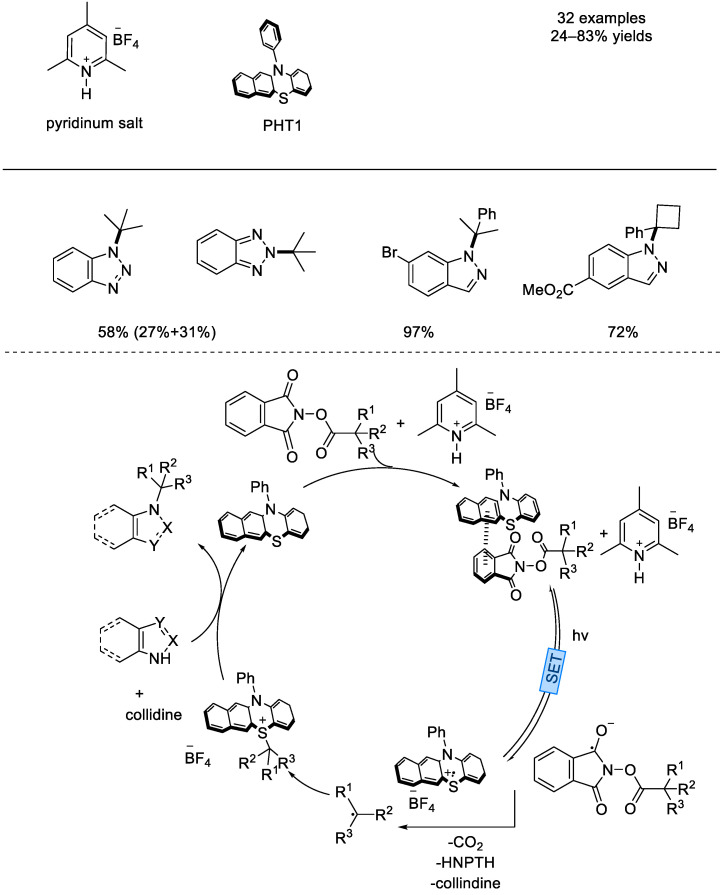
*N*–phenylbenzo[*b*]phenothiazine catalyzed decarboxylative *N*–alkylation of azoles (Ohmiya et al., 2021 [77]).

In 2021, the Ohmiya group reported an organophotoredox–catalyzed radical decarboxylative *N*–alkylation of azoles under visible–light irradiation (Scheme 31) [77]. *N*–phenylbenzo[*b*]phenothiazine was used as the photocatalyst and 2,6–di–*tert*–butylpyridine–derived HBF_4_ salt was used as the additive. Various tertiary or secondary alkyl fragments can add onto the nitrogen atom of azole nucleophiles under mild and transition–metal–free conditions, and the pyridinium additive was found to inhibit the formation of elimination byproducts. It was proposed that the EDA complex of *N*–phenylbenzo[*b*]phenothiazine and NHP esters was the key intermediate.

**Scheme 32:**
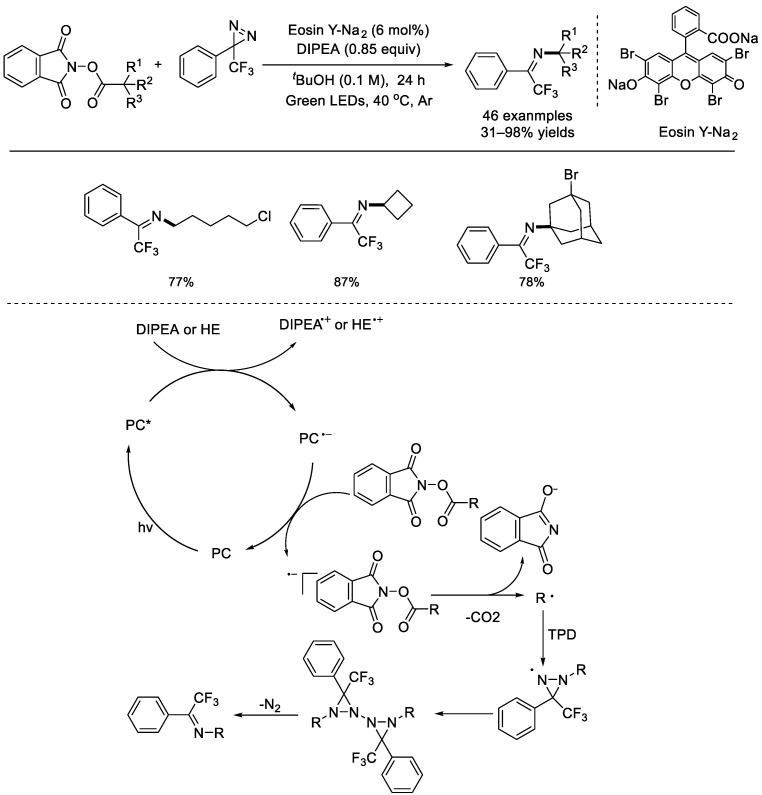
Eosin Y–Na_2_ photocatalyzed decarboxylative C(sp^3^)–N bond–forming reaction with TPD. DIPEA: *N*,*N*–diisopropylethylamine (Liao et al., 2021 [78]).

In 2021, the Liao group discovered a facile, visible light mediated decarboxylative C(sp^3^)–N bond–forming reaction (Scheme 32) [78]. A cheap organic dye, Eosin Y–Na_2_, was used as the organophotocatalyst and commercially available TPD (3–phenyl–3–(trifluoromethyl)–3H–diazirine) was used as the efficient nitrogen source for the decarboxylation amination of NHP esters. This method worked well with various alkyl carboxylic acids, including primary, secondary, and tertiary alkyl carboxylic acids, as well as natural products and drugs. Both imines and diaziridines can be divergently accessible through a single or double nitrogen transfer, respectively. Through altering the reaction conditions, alkylamines and alkylhydrazines can be selectively produced.

## 6. Radical Decarboxylative Coupling with Iodocarboxylates

**Scheme 33:**
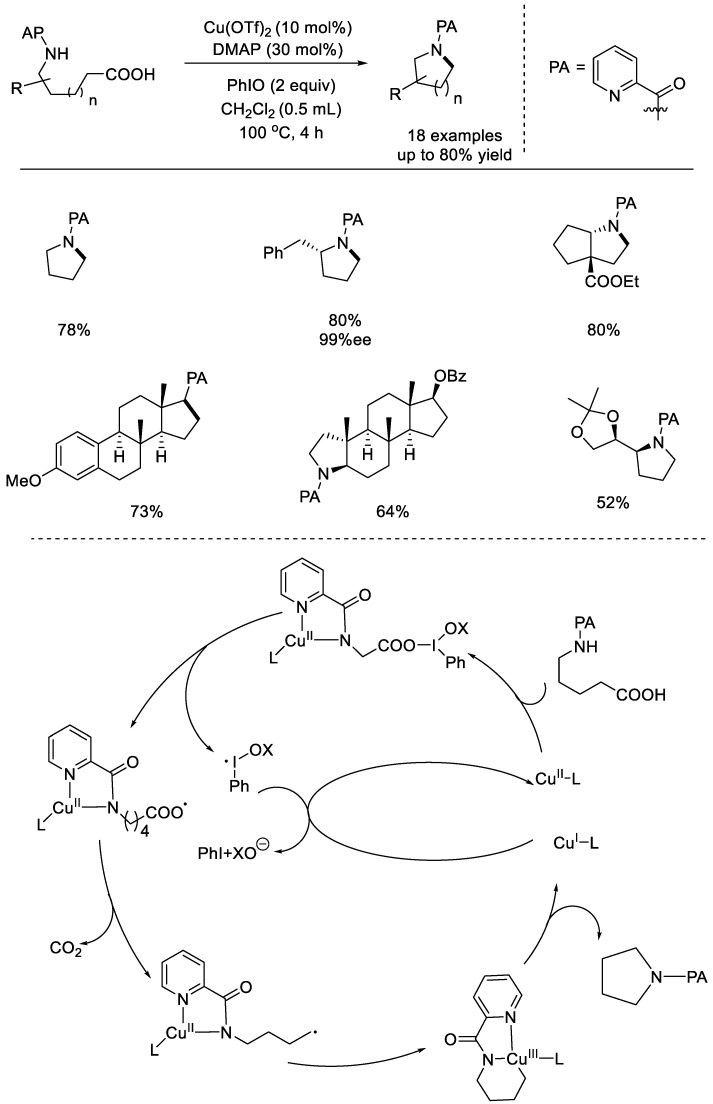
Copper–catalyzed intramolecular decarboxylative cross–coupling C–N bond formation (Fu et al., 2016 [79]).

In 2016, the Fu group reported a copper–catalyzed intramolecular decarboxylative cross–coupling C–N bond formation with nonactivated aliphatic carboxylic acids (Scheme 33) [79]. To control the site–selectivity, a directing group picolinamide (PA) was required. While the formation of 5– and 6–membered heterocycles was successful, the production of 4– or 7–membered heterocycles failed. Notably, modification of complex natural products demonstrated the synthetic value. Homolytic break of the I–O bond produced two radical intermediates, one of which went through a decarboxylation process to generate the alkyl radical. The alkyl radical was oxidatively added to the chelated Cu^II^ species to form a Cu^III^ species. Additionally, the desired product was generated through reductive elimination.

In 2017, the Shi group comprehensively investigated the reaction pathway via DFT calculations [80]. They showed that this reaction involved I^III^–O bond heterolysis, single electron transfer (SET), hydrogen atom transfer (HAT), decarboxylation, proton transfer, and reductive elimination.

**Scheme 34:**
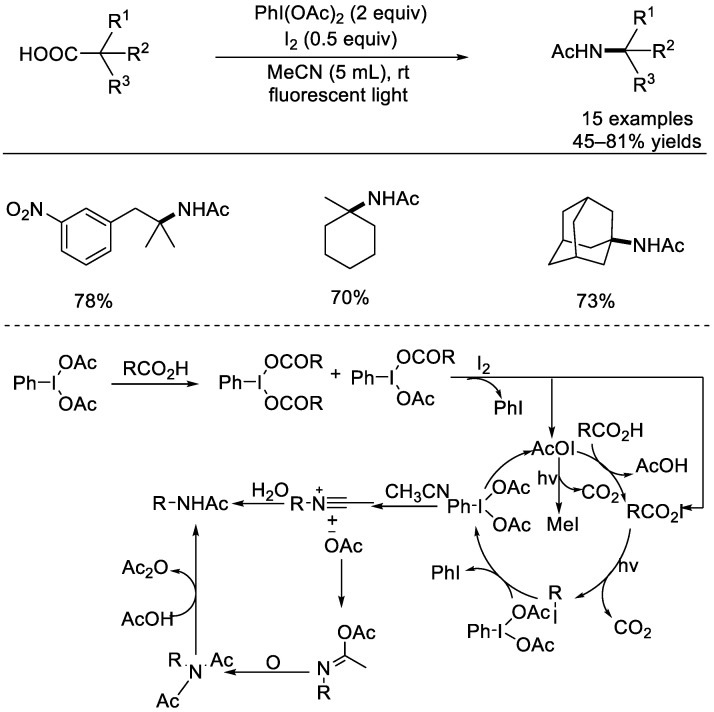
Ritter–type decarboxylation mediated by hypervalent iodine(III) species (Minakata et al., 2017 [81]).

In 2017, the Minakata group reported a Ritter–type decarboxylation mediated using hypervalent iodine(III) (Scheme 34) [81]. The use of PhI(OAc)_2_ in conjunction with molecular iodine promoted this decarboxylate Ritter amination processes of carboxylic acids, and the reactions delivered the tertiary amine derivatives with a range of tertiary alkyl carboxylic acids. Based on control experimental results, the key intermediate might be the RCO_2_I species, which would generate the RI molecule through light–driven decarboxylative iodination. It was supposed that the RI molecule might derive from the radical–radical coupling of the R radical and the iodine atom. The final Ritter–type product can be generated through nucleophilic attack from nitriles.

**Scheme 35:**
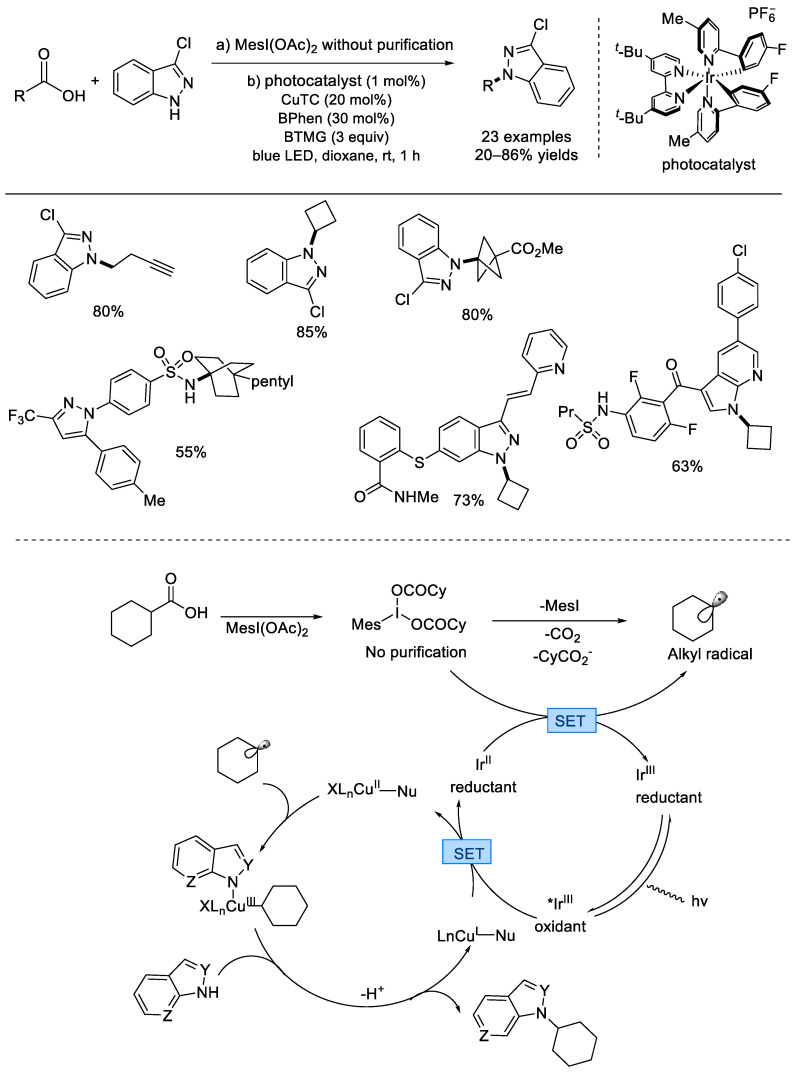
Iridium and copper co–catalyzed decarboxylative C(sp^3^)–N coupling of carboxylic acids and *N*–nucleophilic reagents. CuTC: copper(I) thiophene–2–carboxylate; BPhen: 4,7–diphenyl–1,10–phenanthroline; BTMG: 2–*tert*–butyl–1,1,3,3–tetramethylguanidine; Mes: mesityl (MacMillan et al., 2018 [82]).

In 2018, the MacMillan group elegantly reported an efficient radical decarboxylative C(sp^3^)–N coupling using iridium and copper dual catalysis (Scheme 35) [82]. A variety of *N*–nucleophilic reagents, such as different nitrogen–containing heterocycles, aromatic amines, sulfonamides, and amides that are frequently found in drug molecules afforded the cross–coupled products under mild reaction conditions. Primary, secondary, and tertiary alkyl carboxylic acids were tolerated, which were transformed into iodocarboxylates in situ. Under visible light irradiation, the photocatalyst Ir(III) was excited into a *Ir(III) species, which underwent SET process with L_n_Cu(I)–Nu complex to afford the reduced Ir(II) species and a L_n_Cu(II)–Nu complex. Another SET between Ir(II) species and the in–situ–generated iodocarboxylate produced the regenerated photocatalyst Ir(III) and an alkyl radical after decarboxylation. The latter one can be captured by the L_n_Cu(II)–Nu to form a trivalent Cu(III) complex, and this high–valent–state Cu species was eliminated via rapid reduction to afford the product.

In 2022, the Guan group theoretically discussed the differential between the dual Ir/Cu catalytic system and the system with only a Cu catalyst following McMillan’s work on photo–redox decarboxylative C(sp^3^)–N coupling [83]. It was found that the Ir/Cu dual catalysis was better because the photocatalyst can better satisfy the energy level that matched with SET process, whereas for the Cu single catalysis, a higher energy level gap existed between the Cu complex and the substrate.

**Scheme 36:**
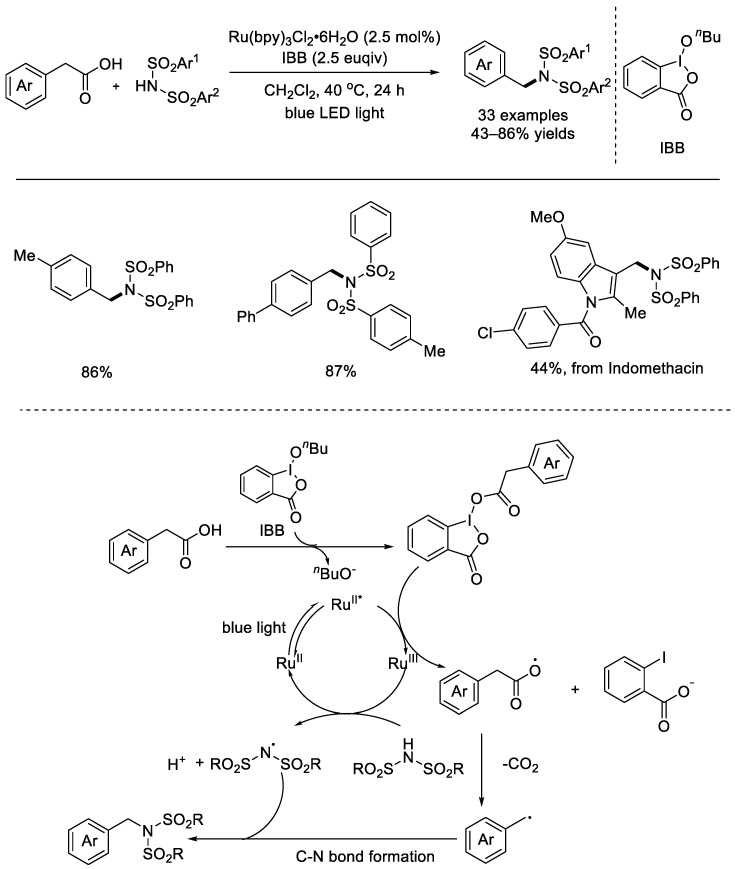
Ru–photo–redox–catalyzed radical decarboxylation of aryl acetic acids to form carbon–nitrogen bonds (Itami et al., 2018 [84]).

In 2018, the Itami group published the photoredox–catalyzed radical decarboxylation of aryl acetic acids to form carbon–nitrogen bonds (Scheme 36) [84]. In the presence of 1–butoxy–1λ^3^–benzo[*d*][1,2]iodaoxol–3(*1H*)–one (IBB), it was not necessary to pre–activate the carboxylic acid coupling partners. The reaction exhibited strong functional group compatibility and a variety of substrates underwent decarboxylation to afford the desired amines in good yields. Moreover, the transformation of pharmaceuticals showed that this method was powerful. Mechanistically, the excited *Ru(II) species underwent SET with the in–situ–generated activated intermediate to afford a Ru(III) species and an alkyl radical upon fragmentation. Subsequently, the Ru(III) species oxidized the imide to provide imidyl radicals and a ground–state Ru(II) species. Then, the final imitation product was formed through radical–radical coupling.

## 7. Radical Decarboxylative Coupling with Oxime Esters

**Scheme 37:**
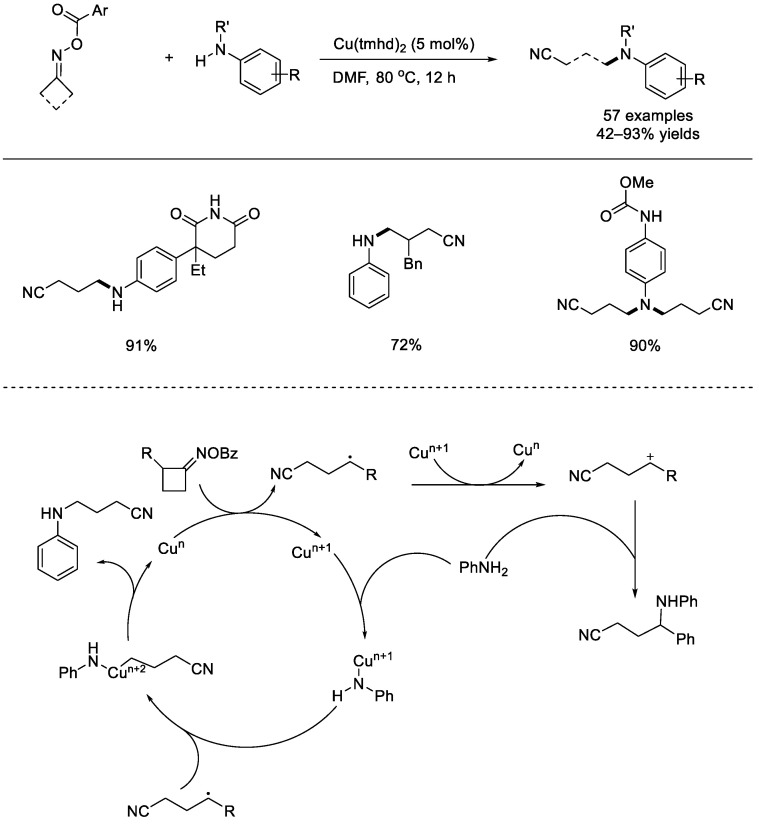
Copper–catalyzed C(sp^3^)–N couplings of cycloketoxime esters by Shi. tmhd: 2,2,6,6–tetramethyl–3,5–heptanedione (Shi et al., 2019 [85]).

In 2019, the Shi group achieved a single–electron–transfer–induced C(sp^3^)–N coupling of cycloketoxime esters (Scheme 37) [85]. Under the copper–catalyzed or synergistic photo–redox/copper–catalyzed reaction conditions, the practical *N*–coupling of cyclic ketoximes with anilines went through C–C bond cleavage and the two powerful and simple methods exhibited high site specificity and substituent compatibility. These amine products containing cyano groups can be used in organic synthesis of functional molecules. Preliminary mechanistic studies indicated that free radical species were involved, and the copper–catalyzed reaction mechanism was depicted. The copper catalyst might undergo a Cu^n^–Cu^n+1^–Cu^n+2^ cycle, and the product was supposed to be generated from the reductive elimination from the high–valent Cu^n+2^ species or S_N_2 reaction between the amine and the benzylic carbocation.

**Scheme 38:**
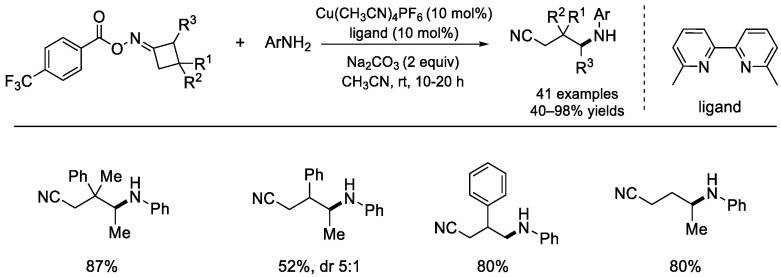
Copper–catalyzed C(sp^3^)–N couplings of cycloketoxime esters by Liu and Fu (Liu et al., 2019 [86]).

In the same year, the groups of Liu [86] and Fu [87] independently reported similar works on copper–catalyzed radical decarboxylative C(sp^3^)–N bond formation with cyclobutanone oxime esters as the alkyl radical sources (Scheme 38). In Liu’s work, 6,6′–dimethyl–2,2′–bipyridine was used as the ligand, while 1,10–phen was applied as the ligand in Fu’s study.

**Scheme 39:**
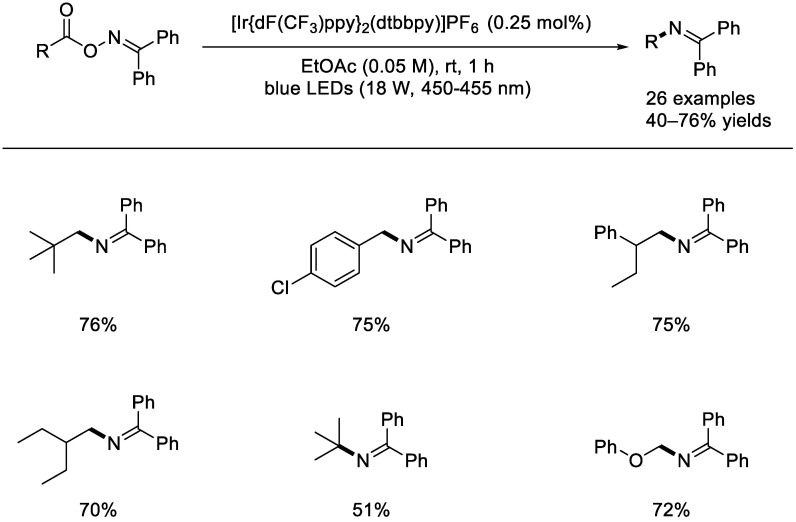
Photocatalytic energy transfer for radical–radical coupling C(sp^3^)–N bonds (Cho et al., 2019 [88]).

In 2019, the Cho group reported an interesting approach for the radical–radical coupling of challenging C(sp^3^)–N bonds exclusively promoted via energy transfer (Scheme 39) [88]. Many oxime ethers decorated with diverse alkyl moiety can afford the corresponding imines in good to high yields. In the presence of the iridium photocatalyst, homolytic cleavage of the N–O bond of an oxime ether happened to form the acyloxyl and imine radical. Based on photoluminescence decay experiments, energy transfer profiles, and DFT calculations, a possible reaction mechanism involving the cross–over coupling of the C and N radicals was proposed [89].

## 8. Radical Decarboxylative Couplings with Diacyl Peroxides

**Scheme 40:**
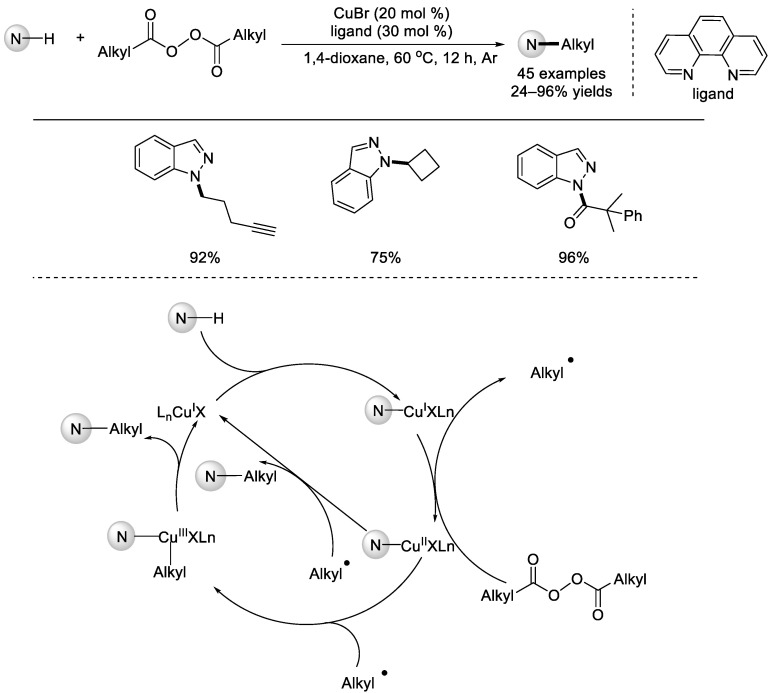
Copper–catalyzed decarboxylative C(sp^3^)–N cross–couplings with diacyl peroxides (Li et al., 2021 [90]).

In 2021, the Li group reported a copper–catalyzed radical decarboxylation for the C(sp^3^)–N cross–coupling of nucleophilic nitrogen reagents and dialkyl diacyl peroxides (Scheme 40) [90]. Various nitrogen nucleophiles, including nitrogen heterocycles, carbazoles, anilines, and sulfonamides, tolerated the conditions. A range of diacyl peroxides were converted into the primary and secondary alkyl radicals. Notably, in some of the cases, the reactions needed to be promoted using [Ir(dtbbpy)(ppy)_2_]PF_6_ under blue LED irradiation at 100 °C. Based on the control experiments, a possible reaction mechanism was proposed. For the reaction mechanism catalyzed by copper, the amino copper(I) species underwent SET with a diacyl peroxide to afford an amino copper(II) species and the resulting alkyl radical. The final *N*–alkyl product can be yielded either through *N*–functionality abstract using the alkyl radical or via reductive elimination from the Cu(III)–alkyl–amino complex.

**Scheme 41:**
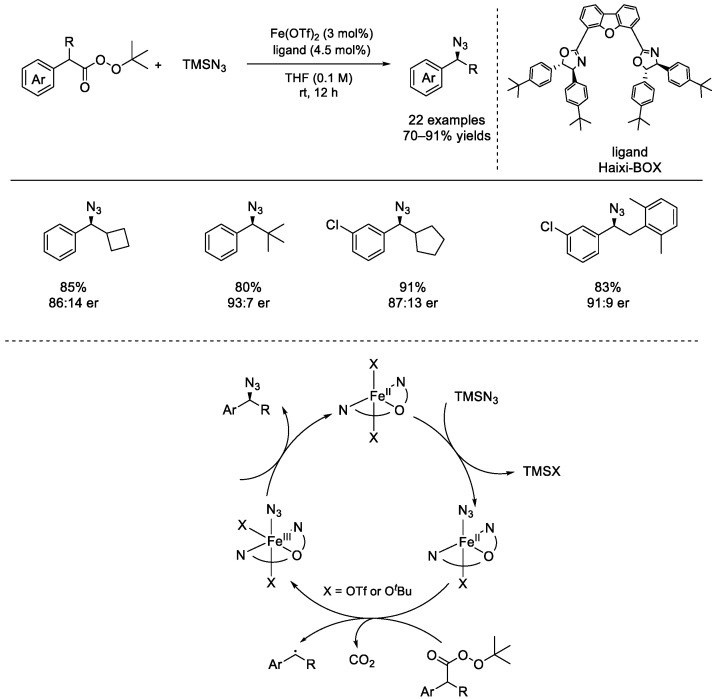
Iron–catalyzed asymmetric azidations of benzyl peresters (Bao et al., 2021 [91]).

In 2021, the Bao group reported the first iron–catalyzed asymmetric azidation of benzylic peresters with trimethylsilyl azide (TMSN_3_) under mild reaction conditions (Scheme 41) [91]. The chiral Haixi–BOX ligand was found to be the best one. Various benzylic peresters performed well and afforded the corresponding enantioenriched azides with high yields and good enantioselectivities. Notably, the generated hydrocarbon radicals were lacking strong interactions. The chiral benzyl azides can be employed in click reactions, phosphoramidation, and reductive amination reactions. Ligand exchange between the *LFe(II) species and TMSN_3_ afforded the *LFe(II)–azide species, which underwent single electron transfer with the perester and would produce the *LFe(III)–azide species and the resulting benzylic carbon radical. An out–sphere group transfer happened to afford the final product and the active catalyst.

## 9. Discussion and Conclusions

In this review article, we have comprehensively discussed the fast–growing developments on radical decarboxylative carbon–nitrogen bond formation from readily available carboxylic acids and their derivatives, such as Barton esters, MPDOC esters, *N*–hydroxyphthalimide esters (NHP esters), oxime esters, aryliodine(III) dicarboxylates, and others. The achievements outlined in this review emphasize the major advances in the field, and the main task is to provide readers with considerable details about this strategy toward modern synthetic chemistry. It is apparent from the classical transition–metal–catalyzed construction of C(sp^2^)–N bonds that this radical decarboxylation process allows the efficient and exciting building of challenging C(sp^3^)–N bonds under suitable reaction conditions, including photocatalysis, light irradiation, transition–metal catalysis, and heat. However, although synthetic potential of this radical decarboxylation has been demonstrated in the synthesis of complex molecules, there is still much to be explored.

(1) Radical decarboxylation often requires the pre–activation of carboxylic acids, which accompanies multiple reaction steps and more reaction waste. From the viewpoint of atom economy and environmental benefits, it is desirable to develop more efficient strategies through directly using carboxylic acids as the reactants.
(2) Another challenging task is the development of enantioselective radical decarboxylation fashion. It would be a great strategy for the synthesis of valuable chiral amines. However, enantiocontrolled radical decarboxylation is still underdeveloped to date. Transition–metal catalysis might be a feasible solution for this task.
(3) Furthermore, it is known that the better understood the mechanism is, the better the synthetic applications will be. Therefore, mechanistic appreciation of radical decarboxylation will be of great value, especially when merged with dual or combined catalytic systems.

Finally, radical decarboxylative carbon–nitrogen bond construction has become a huge field for exploration. We hope that the important historical and recent developments discussed in this review will be a stepping stone for further developments in radical decarboxylation for carbon–nitrogen bond formation both in organic synthesis and medicinal chemistry.

## Data Availability

Not applicable.
